# The role of nutrition education in the pharmacy curriculum using the example of knowledge about the health benefits of nuts

**DOI:** 10.3389/fpubh.2024.1481265

**Published:** 2024-11-25

**Authors:** Ivana Stević, Danijela Milenković, Ana Petrović, Ana Pejčić, Margarita Dodevska, Marija Prvulović Ilić, Nevena Ivanović

**Affiliations:** ^1^Department of Social Pharmacy and Pharmaceutical Legislation, Faculty of Pharmacy, University of Belgrade, Belgrade, Serbia; ^2^Department of Physics and Mathematics, Faculty of Pharmacy, University of Belgrade, Belgrade, Serbia; ^3^Nutritional Studio Ana Petrović, Belgrade, Serbia; ^4^Department of Pharmacology and Toxicology, Faculty of Medical Sciences, University of Kragujevac, Kragujevac, Serbia; ^5^Institute of Public Health of Serbia “Dr. Milan Jovanović Batut”, Belgrade, Serbia; ^6^Department of Bromatology, Faculty of Pharmacy, University of Belgrade, Belgrade, Serbia

**Keywords:** nuts, pharmacy student, attitudes, knowledge, consumption practices, curriculum, nutrition-related course

## Abstract

In recent years, nuts have gained importance because of their nutritional benefits in promoting health and preventing chronic diseases. Despite their recognized role as part of healthy dietary patterns, global consumption is below recommended levels, necessitating an investigation of factors influencing consumption and the impact of nutrition education.

**Objective:**

To investigate the attitudes, knowledge and consumption habits of students in Serbia regarding the consumption of nuts and to examine the role and importance of food- or nutrition-related topics in academic curricula in promoting positive attitudes and habits regarding the consumption of nuts among pharmacy students.

**Methods:**

An electronic questionnaire was used to collect data for this cross-sectional study. A total of 509 responses were collected, including 382 from pharmacy students (75.0%) and 127 from non-pharmacy students (25.0%).

**Results:**

Attitudes toward eating nuts were generally positive, with statistically significant differences found between pharmacy students and non-pharmacy students for 10 statements. The knowledge levels differed, with the mean total number of correct answers in the total sample being 3.9 ± 2.5 (R: 0-11, Mdn: 4) out of 13, and there was a statistically significant difference (*p* < 0.05) between pharmacy students and non-pharmacy students on 8 out of 13 knowledge questions. The study revealed that students obtained information about the health benefits of nuts, mainly from college lectures (51.9%) and mass media (60.9%). More than half of the students (57.8%) expressed a desire for additional information about nuts, which influenced their attitudes significantly more than their level of knowledge.

**Conclusion:**

The results showed that pharmacy students had better knowledge and more positive attitudes toward the consumption of nuts compared to non-pharmacy students. Although completion of nutrition-related courses showed some positive influence, this was not statistically significant for most attitudes and beliefs. These findings underscore the potential value of integrating comprehensive nutrition education into pharmacy curricula, as the combination of knowledge and positive attitudes fostered by pharmacy and nutrition education will enable future health professionals to play a critical role in promoting healthier and sustainable eating habits in the population.

## 1 Introduction

In recent years, the role of nutrition in promoting health and preventing chronic diseases has been increasingly emphasized (1). Owing to their beneficial nutritional properties and potentially positive effects on health, nuts are receiving increasing attention as essential components of a balanced diet (2). Nuts are also an essential part of a plant-based diet that is growing in popularity, not only because of their proven health benefits compared with traditional, animal-based diets but also because of their contribution to the food system’s sustainability and, therefore, planetary health (3–5).

In a broader context, nuts are a nutrient-rich, energy-dense category of plant food and, together with other plant foods such as legumes, seeds, and whole grains, are an integral part of the Mediterranean diet, known for their health-promoting effects (6, 7). The results of numerous intervention and prospective cohort studies have shown that the health benefits of nut consumption are reflected mainly in the reduction in the risk of cardiovascular disease and all-cause mortality, including lowering systolic blood pressure, as well as reducing the incidence of metabolic syndrome and diabetes (6, 8–10). In addition, data from the literature indicate an inverse correlation between nut consumption, body mass index (BMI), and waist circumference (11). Therefore, the American Heart Association and the WHO officially recommend the consumption of nuts as part of a healthy and balanced diet, making them an important part of dietary recommendations for health promotion and non-communicable disease prevention (12, 13). Recommendations for the consumption of nuts are also found in numerous national food-based dietary guidelines (FBDGs), where they are generally categorized together with seeds in the protein food group as an alternative to foods of animal origin, such as meat, eggs, and seafood (14). For example, in the Dietary Guidelines for Americans 2020–2025, nuts are included as nutrient-dense food and a source of protein, and the consumption of 5 ounces of nuts per week, including seeds, is recommended (15). Nuts are also included as an alternative to red meat in the Eat-Lancet healthy reference diet, which recommends the consumption of 50 g of nuts (16).

In addition to their importance as a source of protein in the diet, nuts are important dietary sources of monounsaturated and polyunsaturated fatty acids, including essential fatty acids, phytosterols, fiber, and polyphenols, with favorable mineral compositions, especially those with low sodium contents (17, 18). The favorable composition of fatty acids and the suitable ratio of unsaturated to saturated fatty acids are commonly responsible for the health effects of eating nuts. The health effects of nut consumption are also confirmed by the health claims approved by the Food and Drug Agency (FDA) and the European Food Safety Agency (EFSA), which refer to the beneficial effects of consuming 43 g (approximately 1.5 recommended servings) of nuts on reducing the risk of heart disease (19) and the importance of consuming 30 g of walnuts per day as part of a balanced diet to improve endothelium-dependent vasodilation (20).

Despite the well-documented benefits of nuts, studies suggest that their consumption is still suboptimal in many population groups. According to surveys conducted in various countries, population-level rates for whole nut consumption are between 2 and 7% of participants, with an average population intake of 2.2–3 g/day (2, 11, 21). Several studies have pointed to the role of socioeconomic and educational factors in nut consumption. People with higher levels of education and socioeconomic status were found to be more likely to consume nuts, but one of the barriers to nut consumption was their price (11, 22). Considering all this, especially the health importance of nut consumption on the one hand and the low consumption in different countries worldwide compared with the guidelines on the other hand, it is important to examine the factors as well as the obstacles that are important for regular nut consumption.

Since education, especially in health-related subjects, can influence dietary habits, this study specifically examines the attitudes, knowledge and consumption habits of students in Serbia, with a focus on pharmacy students. Furthermore, this work aims to assess the influence of food and nutrition-related topics in academic curricula, especially in pharmacy education, on the development of positive attitudes and health-promoting dietary habits, which include regular consumption of nuts. This focus on pharmacy students is critical as they will play an important role in public health and nutrition counseling as future health professionals.

## 2 Materials and methods

An electronic survey was conducted via a questionnaire specially created for this cross-sectional study.

The minimum sample size was determined according to the Cochran method for large populations (23). Using a confidence level of 95%, a margin of error of 5%, and a conservative proportion estimate of *p* = 0.5, we calculated an original sample size of 385. To check our calculation, we used the Qualtrics sample size calculator as an additional tool (24).

The study consisted of two phases: (a) development and testing of the questionnaire and (b) conducting research on a specific population.

- The questions in the questionnaire were selected by screening the relevant literature that was publicly available (e.g., PubMed and *Google Scholar*). Questions that were irrelevant or otherwise inappropriate for this study were excluded by mutual agreement between the researchers. To ensure the clarity, comprehensibility and relevance of the questions to the aims of the study, the questionnaire was pre-tested using Google Forms in a small group of experts and in a sample of 10 students who were not participating in the main study. The feedback from this pre-test led to minor revisions to improve the readability and relevance of the questions.The final questionnaire consisted of 5 groups of questions: (i) questions related to sociodemographic characteristics (*N* = 24); (ii) questions related to sources of information on the nutritional value of nuts used by respondents (*N* = 4); (iii) statements related to attitudes regarding the health effects and nutritional importance of nuts (*N* = 22); (iv) questions related to students’ knowledge of the nutritional value of nuts (*N* = 13); and (v) questions related to the dietary practices of the respondents (which nuts they most often consume, for what reason, how often and in what form) (*N* = 19).
- A study on the student population of the Republic of Serbia was conducted from November 2023–March 2024. Using the snowball technique, the final questionnaire from Phase 1 was disseminated via the Google Forms link through social networks, e-mail, and different platforms (Viber, WhatsApp, LinkedIn). The inclusion criteria for participation in the research were that the respondent was a student in the Republic of Serbia, had given informed consent to participate, and had not filled out the same questionnaire before. The data collection was anonymous, voluntary, and without requests for the respondents’ personal data. The study was approved by the Ethics Committee of the Faculty of Pharmacy, University of Belgrade.

Statistical analysis was performed via IBM SPSS Statistics, version 29. Descriptive statistics were calculated to describe the sample characteristics. The categorical variables are presented as frequencies and percentages, and the continuous variables are reported as the mean value, standard deviation, median (Mdn) and total range (R). The chi-square test was used in contingency table analysis to confirm a statistically significant difference between groups. The corresponding values of Pearson’s chi-square statistic or Fisher’s exact probability indicator were calculated depending on the sample size in the observed table. The significance threshold was set to 0.05.

## 3 Results

### 3.1 Characteristics of the participants

The total number of respondents was 513, of which 509 were used for the final statistical analysis (4 were excluded because they did not fulfill the inclusion criterion of being a student), with a majority of female respondents (86.1%). The average age of all the participants was 23.1 ± 2.5 years (R: 18–35, Mdn: 23.0), with an average BMI of 21.8 ± 3.0 kg/m^2^ (R: 15.9–43.7, Mdn: 21.3). In terms of BMI, 80.5% of the participants had normal weight, 7.9% were underweight, and 11.7% were overweight or obese. There was a statistically significant difference in the distribution of respondents across BMI categories, including underweight, normal weight, overweight, and obese [*χ*^2^ (*N* = 507, df = 4) = 1178.335, *p* < 0.001]. Additional data and results concerning age, height, weight, and BMI are provided in the Supplementary material.

The questionnaire was completed by students from faculties of various profiles (biomedical sciences, technical sciences and social-humanistic sciences). The participants studied at different universities in the Republic of Serbia. All the respondents were divided into two categories: studying pharmacy (75%) or any other faculty (25%), keeping our objectives in mind, to perform an additional analysis comparing participants who had food- or nutrition-related subjects in the curricula with those who did not. The group of “non-pharmacy students” includes students from more than 20 different faculties (e.g., humanities, technical faculties, agriculture, biology, dentistry, horticulture, faculty of nursing…). None of the students from the non-pharmacy group stated that they had nutrition-related subjects in their curriculum or had taken an exam in this area. Keeping our objectives in mind, we also performed an additional analysis comparing participants who had food- or nutrition-related subjects in the curricula with those who did not.

More than 80% were nonsmokers, whereas in the “smoker” category, only 1.2% smoked more than 20 cigarettes per day. Two-thirds of the respondents consumed alcohol, whereas half of the respondents consumed alcohol rarely, once or twice a month. Approximately 1% of the respondents consumed alcohol more than three times a week. Most students consumed one to two glasses of wine (26.3%), followed by one to two glasses of beer (18.7%). Interestingly, more than 3 glasses of any type of drink (spirit drink, beer, or wine) are consumed 1–2 times a month by 74% of the respondents.

Most respondents could classify their dietary practices under one of the 7 offered categories (Table 1) or write an answer if none of the proposed categories fit. The additional answers represent a combination of the already proposed categories, e.g., the diet is regular but with a reduced intake of dairy products, sugar, or carbohydrates. Interestingly, vegan, vegetarian, and gluten-free diet types were still very rarely represented in the respondents’ answers. The sociodemographic characteristics and habits of the respondents are provided in Table 1.

**Table 1:** Sociodemographic characteristics and habits of the respondents.

Variable	Answer	*N* (%)
Gender	Male	71 (13.9)
	Female	438 (86.1)
Study program	Pharmacy/Pharmacy - medical biochemist	382 (75.0)
	Others	127 (25.0)
Study year	1	49 (9.6)
	2	59 (11.6)
	3	102 (20.0)
	4	97 (19.1)
	5	202 (39.7)
Smoke status	Smoker	71 (13.9)
	Non-smoker	419 (82.3)
	Ex-smoker	19 (3.7)
Number of cigarettes per day	No answer	2 (0.4)
	<10	32 (6.3)
	10–20	34 (6.7)
	More than 20	6 (1.2)
	Nonsmoker	435 (85.5)
Alcohol status	Consume	341 (67.0)
	Not consume	168 (33.0)
Alcohol consumption frequency	Daily	1 (0.2)
	More than 3 times in a week	4 (0.8)
	2–3 times in a week	19 (3.7)
	Once weekly	61 (12.0)
	1–2 times in a month	256 (50.3)
	Not consume	168 (33.0)
Dietary preferences	Regular	409 (80.4)
	Vegetarian (no meat consumption, but consuming eggs and milk products)	12 (2.3)
	Vegan (no food of animal origin)	2 (0.4)
	Diet (low calorie intake)	28 (5.5)
	No gluten food	3 (0.6)
	Reduced carbs and sugar intake due to insulin resistance or diabetes	29 (5.7)
	Other	26 (5.1)
Food allergy	Yes	34 (6.7)
	No	475 (93.3)

In our study, 34 (6.7%) respondents were allergic to one or more foods. The same number of respondents confirmed that they had an allergic reaction that required medical attention/hospitalization. However, out of these 34 affirmative answers, 28 respondents gave a negative answer to the question about food allergies, i.e., in the mentioned cases, the allergic reactions that required medical attention were not necessarily caused by food allergies. An analysis of the responses revealed that milk and milk products caused the highest percentage of allergic reactions (as much as 25.71%). Some of the allergens, such as soy, sesame, shallot, and celery, which were listed above, were not recognized as causative agents by our study participants. Almost 15% of the respondents who reported registered allergies listed kiwifruit as an allergen, whereas almost 12% listed grains containing gluten, nuts, or peanuts as allergens.

When asked whether they have certain knowledge about the health effects of nuts, 67.4% answered affirmatively, 9% answered negatively, and 23.6% said they did not know. Interestingly, 72.8% of the pharmacy students said that they had knowledge, while this percentage was lower (50.4%) among respondents who studied another faculty. Additionally, approximately 48% of the respondents who did not have subjects related to food- or nutrition-related topics in the curricula stated that they had knowledge about health effects.

A total of 44.2% of the respondents did not take the subjects’ food or nutrition-related topics (represented as courses: Bromatology or Nutrition) or did not have them in the study program at the time of the survey. Of the remaining 55.8%, slightly more than half stated that they had received enough information on these topics. Surprisingly, as many as 78 respondents (27.5% of those who took the subjects) believed that they did not receive enough information in the nutrition-related courses, whereas the remaining respondents did not remember how much information they learned in these courses. Encouragingly, three out of four respondents (74.7%) were aware of the importance of this topic and stated that they would like to receive additional information about the importance of nuts.

### 3.2 Attitudes regarding health benefits of nuts

**Figure 1:**
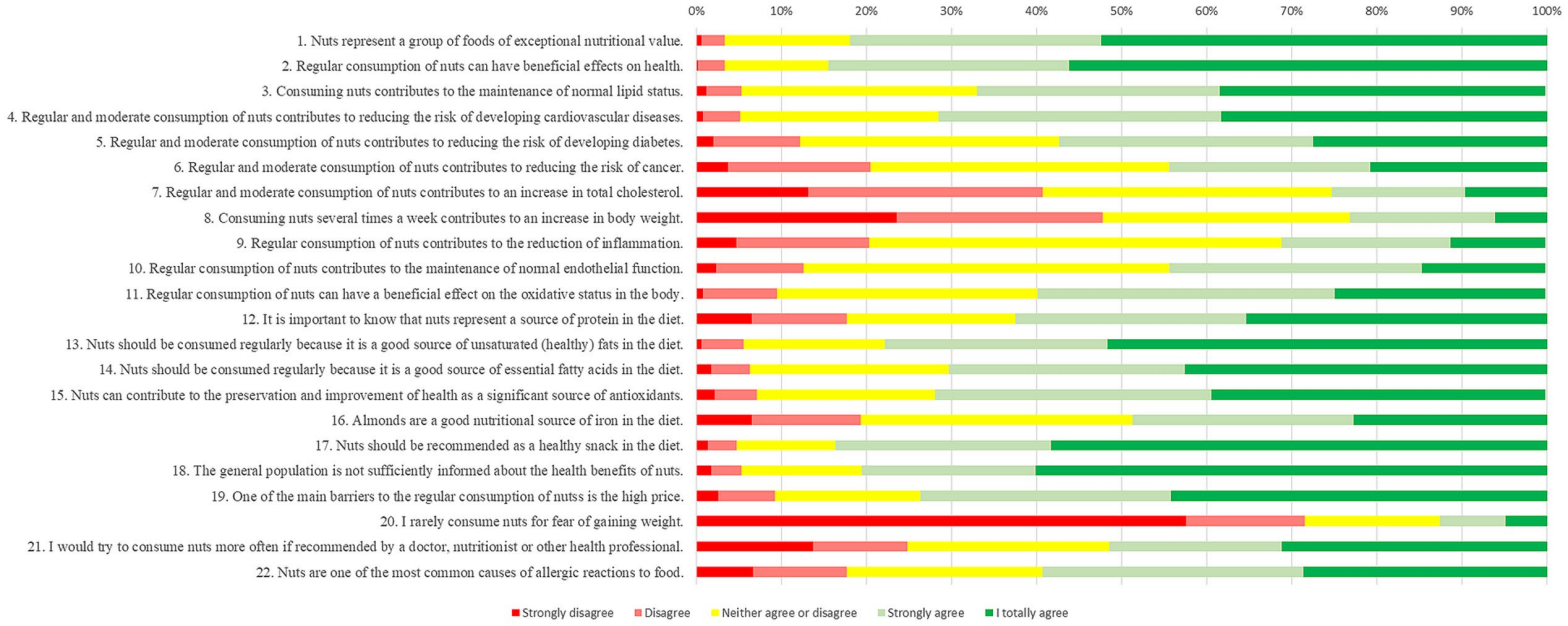
Attitudes regarding the health benefits and nutritional importance of nuts.

In the third part of the questionnaire, the respondents expressed their attitudes regarding the health benefits and nutritional importance of nuts. There were 22 statements for which the respondents had to mark a number on a scale of 1–5 (1: totally disagree to 5: totally agree) in the degree to which the statement best reflected their opinion. Figure 1 shows the attitudes of students regarding the health benefits and nutritional importance of nuts.

The respondents had positive attitudes toward the majority of the statements (15/22). The only statement with which more than 70% of the respondents disagreed was “I rarely consume nuts for fear of gaining weight.”

The results from assessing the faculty’s impact on the answers to the statements about the health benefits and nutritional importance of nuts are presented in Table 2. All the respondents were grouped into two categories: pharmacy students and students in other faculties (non-pharmacy students). For the 10 statements, there was a statistically significant difference in attitudes between pharmacy and non-pharmacy students.

**Table 2:** The influence of attending the faculty of pharmacy on perceptions and beliefs regarding the health benefits and nutritional importance of nuts.

Statement	Students	Strongly disagree *N* (%)	Disagree *N* (%)	Neither agree or disagree *N* (%)	Agree *N* (%)	Strongly agree *N* (%)	Stat.^†^	*p*-value
1. Nuts represent a group of foods of exceptional nutritional value.	Pharmacy *N* = 382	1 (0.3)	10 (2.6)	50 (13.1)	121 (31.7)	200 (52.4)	8.219^f^	0.071
	Non-pharmacy *N* = 127	2 (1.6)	4 (3.1)	25 (19.7)	29 (22.8)	67 (52.8)		
2. Regular consumption of nuts can have beneficial effects on health.	pharmacy *N* = 382	0 (0.0)	7 (1.8)	38 (9.9)	112 (29.3)	225 (58.9)	18.410^f^	0.001*
	Non-pharmacy *N* = 127	1 (0.8)	9 (7.1)	24 (18.9)	32 (25.2)	61 (48.0)		
3. Consuming nuts contributes to the maintenance of normal lipid status.	Pharmacy *N* = 381	3 (0.8)	13 (3.4)	93 (24.4)	120 (31.5)	152 (39.9)	15.629	0.004*
	Non-pharmacy *N* = 127	3 (2.4)	8 (6.3)	48 (37.8)	25 (19.7)	43 (33.9)		
4. Regular and moderate consumption of nuts contributes to reducing the risk of developing cardiovascular diseases.	Pharmacy *N* = 382	1 (0.3)	12 (3.1)	88 (23.0)	126 (33.0)	155 (40.6)	12.440	0.015*
	Non-pharmacy *N* = 127	3 (2.4)	10 (7.9)	31 (24.4)	43 (33.9)	40 (31.5)		
5. Regular and moderate consumption of nuts contributes to reducing the risk of developing diabetes.	Pharmacy *N* = 382	6 (1.6)	36 (9.4)	109 (28.5)	123 (32.2)	108 (28.3)	7.125	0.128
	Non-pharmacy *N* = 127	4 (3.1)	16 (12.6)	46 (36.2)	29 (22.8)	32 (25.2)		
6. Regular and moderate consumption of nuts contributes to reducing the risk of cancer.	Pharmacy *N* = 382	11 (2.9)	62 (16.2)	134 (35.1)	93 (24.3)	82 (21.5)	3.878	0.426
	Non-pharmacy *N* = 127	8 (6.3)	23 (18.1)	45 (35.4)	27 (21.3)	24 (18.9)		
7. Regular and moderate consumption of nuts contributes to an increase in total cholesterol.	Pharmacy *N* = 382	47 (12.3)	107 (28.0)	129 (33.8)	61 (16.0)	38 (9.9)	1.248	0.874
	Non-pharmacy *N* = 127	20 (15.7)	33 (26.0)	44 (34.6)	19 (15.0)	11 (8.7)		
8. Consuming nuts several times a week contributes to an increase in body weight.	Pharmacy *N* = 382	86 (22.5)	92 (24.1)	117 (30.6)	65 (17.0)	22 (5.8)	2.286	0.685
	Non-pharmacy *N* = 127	34 (26.8)	31 (24.4)	31 (24.4)	22 (17.3)	9 (7.1)		
9. Regular consumption of nuts contributes to the reduction of inflammation.	Pharmacy *N* = 381	15 (3.9)	61 (16.0)	174 (45.7)	85 (22.3)	46 (12.1)	10.446	0.033*
	Non-pharmacy *N* = 127	9 (7.1)	18 (14.2)	73 (57.5)	16 (12.6)	11 (8.7)		
10. Regular consumption of nuts contributes to the maintenance of normal endothelial function.	Pharmacy *N* = 381	9 (2.4)	35 (9.2)	153 (40.2)	126 (33.1)	58 (15.2)	10.915	0.027*
	Non-pharmacy *N* = 127	3 (2.4)	17 (13.4)	66 (52.0)	25 (19.7)	16 (12.6)		
11. Regular consumption of nuts can have a beneficial effect on the oxidative status in the body.	Pharmacy *N* = 381	0 (0.0)	30 (7.9)	110 (28.9)	140 (36.7)	101 (26.5)	17.820	0.001*
	Non-pharmacy *N* = 127	4 (3.1)	14 (11.0)	46 (36.2)	38 (29.9)	25 (19.7)		
12. It is important to know that nuts represent a source of protein in the diet.	Pharmacy *N* = 382	21 (5.5)	40 (10.5)	76 (19.9)	110 (28.8)	135 (35.3)	4.622	0.330
	Non-pharmacy *N* = 127	12 (9.4)	17 (13.4)	25 (19.7)	28 (22.0)	45 (35.4)		
13. Nuts should be consumed regularly because it is a good source of unsaturated (healthy) fats in the diet.	Pharmacy *N* = 382	1 (0.3)	18 (4.7)	58 (15.2)	100 (26.2)	205 (53.7)	6.200	0.174
	Non-pharmacy *N* = 127	2 (1.6)	7 (5.5)	27 (21.3)	33 (26.0)	58 (45.7)		
14. Nuts should be consumed regularly because it is a good source of essential fatty acids in the diet.	Pharmacy *N* = 382	6 (1.6)	15 (3.9)	77 (20.2)	108 (28.3)	176 (46.1)	12.755	0.012*
	Non-pharmacy *N* = 127	3 (2.4)	8 (6.3)	42 (33.1)	33 (26.0)	41 (32.3)		
15. Nuts can contribute to the preservation and improvement of health as a significant source of antioxidants.	Pharmacy *N* = 381	3 (0.8)	20 (5.2)	69 (18.1)	130 (34.1)	159 (41.7)	23.428	<0.001*
	Non-pharmacy *N* = 127	8 (6.3)	5 (3.9)	38 (29.9)	35 (27.6)	41 (32.3)		
16. Almonds are a good nutritional source of iron in the diet.	Pharmacy *N* = 382	24 (6.3)	53 (13.9)	121 (31.7)	96 (25.1)	88 (23.0)	2.036	0.732
	Non-pharmacy *N* = 127	9 (7.1)	12 (9.4)	42 (33.1)	36 (28.3)	28 (22.0)		
17. Nuts should be recommended as a healthy snack in the diet.	Pharmacy *N* = 382	2 (0.5)	11 (2.9)	41 (10.7)	102 (26.7)	226 (59.2)	11.307	0.023*
	NON-pharmacy *N* = 127	5 (3.9)	6 (4.7)	18 (14.2)	27 (21.3)	71 (55.9)		
18. The general population is not sufficiently informed about the health benefits of nuts.	Pharmacy *N* = 382	4 (1.0)	12 (3.1)	49 (12.8)	80 (20.9)	237 (62.0)	8.196	0.082
	Non-pharmacy *N* = 127	5 (3.9)	6 (4.7)	23 (18.1)	24 (18.9)	69 (54.3)		
19. One of the main barriers to the regular consumption of nuts is the high price.	Pharmacy *N* = 382	9 (2.4)	25 (6.5)	62 (16.2)	120 (31.4)	166 (43.5)	3.100	0.546
	Non-pharmacy *N* = 127	4 (3.1)	9 (7.1)	25 (19.7)	30 (23.6)	59 (46.5)		
20. I rarely consume nuts for fear of gaining weight.	Pharmacy *N* = 382	222 (58.1)	56 (14.7)	59 (15.4)	27 (7.1)	18 (4.7)	1.676	0.798
	Non-pharmacy *N* = 127	71 (55.9)	15 (11.8)	22 (17.3)	12 (9.4)	7 (5.5)		
21. I would try to consume nuts more often if recommended by a doctor, nutritionist or other health professional.	Pharmacy *N* = 382	47 (12.3)	40 (10.5)	90 (23.6)	82 (21.5)	123 (32.2)	4.356	0.362
	Non-pharmacy *N* = 127	23 (18.1)	16 (12.6)	31 (24.4)	21 (16.5)	36 (28.3)		
22. Nuts are one of the most common causes of allergic reactions to food.	Pharmacy *N* = 382	16 (4.2)	39 (10.2)	85 (22.3)	122 (31.9)	120 (31.4)	20.267	<0.001*
	Non-pharmacy *N* = 127	18 (14.2)	17 (13.4)	32 (25.2)	34 (26.8)	26 (20.5)		

^†^Chi-square test, ^f^Fisher’s exact test, *statistically significant difference.

### 3.3 Knowledge related to nuts and their nutritional composition

The fourth part of the questionnaire assessed students’ knowledge through 13 specific questions. All the questions were closed type, where the answer “I do not know” was also an option.

The mean total number of correct answers in the total sample was 3.9 ± 2.5 (R: 0–11, Mdn: 4), whereas in the population of students who successfully passed the exams (food or nutrition-related subjects), it was 4.89 ± 2.4 (R: 0–11, Mdn: 5). A statistically significant difference was found in the number of correct, incorrect and unknown answers between the groups of students who passed the dietary exams and those who did not pass those exams (χ^2^(*N* = 509, df = 11) = 84.935, *p* < 0.001). Descriptive statistics describing the numbers of correct, incorrect and “I do not know” answers are given in the Supplementary material.

Interestingly, the maximum number of correct answers was 11, meaning that no respondent correctly answered 12 or 13. The lowest percentage of answers, “I do not know,” appeared in the first question (26.2%), indicating that more than a quarter of the students did not give the answer (either correct or incorrect) to at least one of the 13 questions. Interestingly, 26 out of 509 respondents answered “I do not know” to all questions, indicating that 5.1% of students either did not know the answers to all questions or did not feel confident enough to answer them. Unfortunately, 5 of the 249 students who took/passed both exams had 0 correct answers.

**Figure 2:**
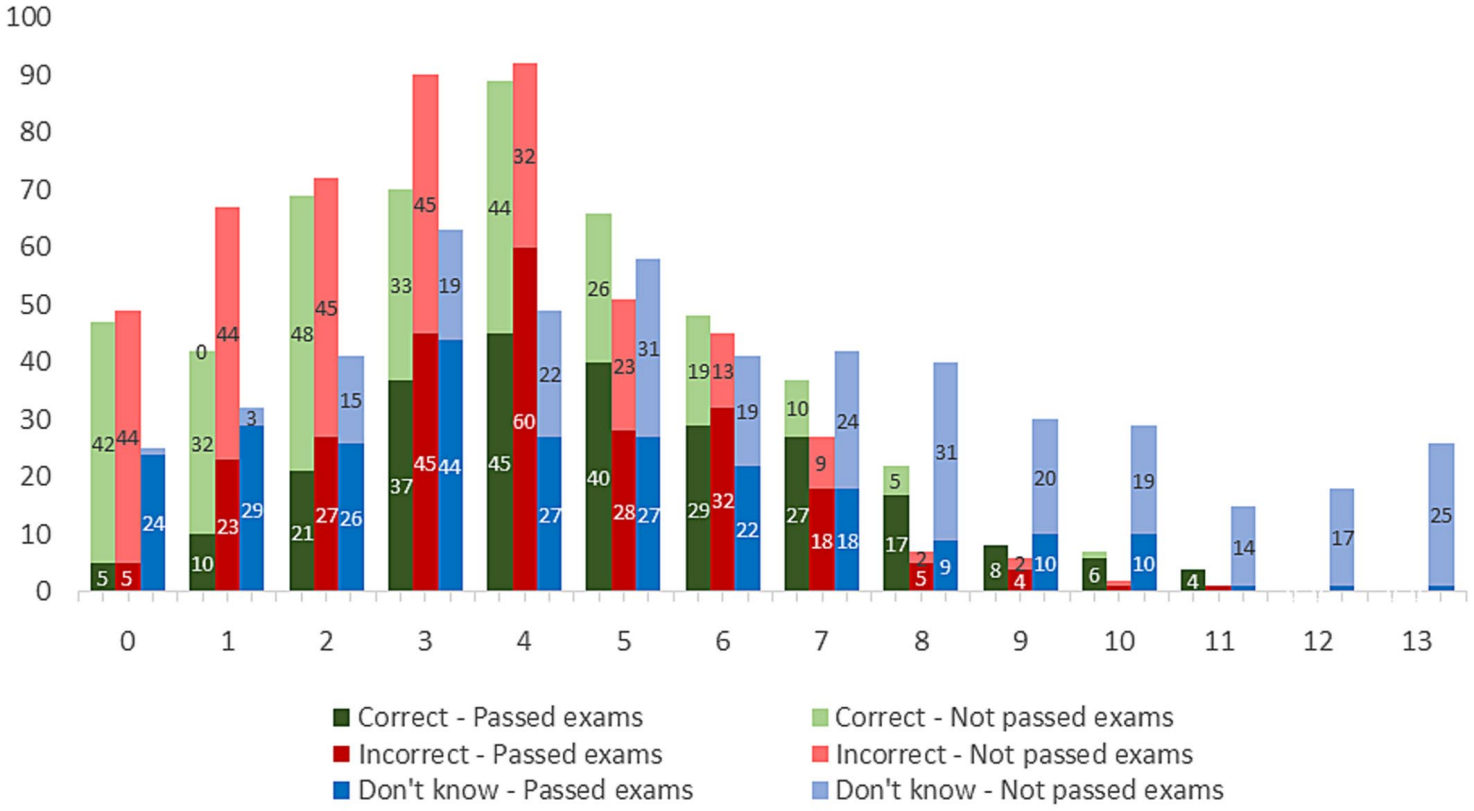
The frequency of the total number of correct/incorrect/unknown answers.

Figure 2 shows the frequency of the total number of correct, incorrect and unknown answers among all students, represented in green, red and blue bars, respectively. The frequencies of the total numbers in the subgroup of students who previously passed the nutrition tests are highlighted with a darker color, while a lighter color symbolizes the subgroup of students who did not pass the nutrition tests. The horizontal axis represents the specific number of responses out of a maximum of 13, i.e., category 0 shows the frequency of students who had 0 correct (green bar), 0 incorrect (red bar) and 0 unknown responses (blue bar), etc. Interestingly, the maximum number of correct answers was 11, which means that no respondent answered 12 or 13 correctly. The largest number of students who passed the exams had 4 correct answers (45 out of 249), while the largest number of students who failed the exams had 2 correct answers (48 out of 260).

Table 3 shows the students’ knowledge of nuts, where a comparison was also made between pharmacy and non-pharmacy students. Individual tables (pharmacy, non-pharmacy) are given in the Supplementary material, as well as the impact of the passed exams (courses related to food or nutrition-related topics in the curricula) on knowledge. For 8 out of the 13 questions, there was a statistically significant difference (*p* < 0.05) in knowledge related to nuts between pharmacy and non-pharmacy students.

**Table 3:** Influence of studying the faculty of pharmacies on students’ knowledge of nuts.

Question	Answer	Pharmacy students (*N* = 382)		Non-pharmacy students (*N* = 127)		Test	
		*N*	%	*N*	%	Stat.^†^	*P*
1. In the dietary recommendations (nutrition pyramid), nuts are classified together with the group of foods:	Cereals	109	28.5	32	25.2	10.926^f^	0.079
	Milk and milk products	10	2.6	4	3.1		
	Fruits	87	22.8	20	15.7		
	Vegetables	4	1.0	0	0.0		
	*meat and eggs*	54	14.1	13	10.2		
	Oils	30	7.9	12	9.4		
	I do not know	88	23.0	46	36.2		
2. Nuts are an important food group in the Mediterranean diet:	*Yes*	250	65.4	50	39.4	31.168	<0.001*
	No	27	7.1	8	6.3		
	I do not know	105	27.5	69	54.3		
3. Nuts are an important food group in the DASH diet:	*Yes*	135	35.3	15	11.8	27.524	<0.001*
	No	15	3.9	3	2.4		
	I do not know	232	60.7	109	85.8		
4. Nuts definitely are not a source of which mineral in the diet?	Selenium	8	2.1	2	1.6	15.200	0.009*
	*Iodine*	149	39.0	32	25.2		
	Magnesium	26	6.8	5	3.9		
	Iron	24	6.3	7	5.5		
	Zinc	24	6.3	6	4.7		
	I do not know	151	39.5	75	59.1		
5. Nuts are a rich source of which vitamin in the diet?	Vitamin A	16	4.2	8	6.3	21.955	0.001*
	Vitamin C	12	3.1	2	1.6		
	*Vitamin E*	169	44.2	31	24.4		
	Vitamin D	14	3.7	5	3.9		
	Vitamin B12	66	17.3	22	17.3		
	I do not know	105	27.5	59	46.5		
6. Which of the nuts representatives is considered a good source of dietary selenium?	Almond	42	11.0	10	7.9	7.581	0.179
	Hazelnut	7	1.8	1	0.8		
	*Brazilian nut*	98	25.7	23	18.1		
	Walnut	21	5.5	5	3.9		
	Cashew	51	13.4	18	14.2		
	I do not know	163	42.7	70	55.1		
7. Nuts are primarily a source of which polyunsaturated fatty acid?	EPA	37	9.7	2	1.6	30.869	<0.001*
	DHA	24	6.3	4	3.1		
	*Linoleic acid*	34	8.9	8	6.3		
	Alpha-linolenic acid	56	14.7	7	5.5		
	Arachidonic acid	30	7.9	5	3.9		
	I do not know	201	52.6	101	79.5		
8. Which of nuts has the highest content of saturated fatty acids and is therefore the least recommended in the diet compared to other stone fruits?	Peanuts	167	43.7	52	40.9	3.348	0.656
	Almond	23	6.0	7	5.5		
	Walnut	20	5.2	3	2.4		
	Hazelnut	5	1.3	3	2.4		
	*Cashew*	42	11.0	14	11.0		
	I do not know	125	32.7	48	37.8		
9. There is an approved health claim for walnuts that reads: “Walnuts contribute to improving the elasticity of blood vessels.” The stated health statement refers to the daily intake of walnuts in which of the stated quantities?	10 g	33	8.6	9	7.1	9.539^f^	0.128
	15 g	63	16.5	13	10.2		
	*30 g*	87	22.8	25	19.7		
	50 g	23	6.0	14	11.0		
	100 g	8	2.1	3	2.4		
	More than 100 g	0	0.0	1	0.8		
	I do not know	168	44.0	62	48.4		
10. Thermal processing of nuts impacts health effect, in which direction:	*Lowers*	209	54.7	52	40.9	9.095	0.027*
	Increases	15	3.9	4	3.1		
	Do not change	47	12.3	17	13.4		
	I do not know	111	29.1	54	42.5		
11. Thermal processing of nuts impacts allergenicity, in which direction:	Lowers	83	21.7	13	10.2	12.709	0.005*
	Increases	30	7.9	9	7.1		
	*do not change*	111	29.1	32	25.2		
	I do not know	73	41.4	73	57.5		
12. According to its composition and health effects, peanuts can be counted as a nut, even though it belongs to the leguminous family:	*True*	188	49.2	43	33.9	10.553	0.005*
	False	31	8.1	9	7.1		
	I do not know	163	42.7	75	59.1		
13. By what characteristic does the chestnut significantly differ from other types of nuts?	Low content of mineral substances	10	2.6	6	4.7	4.047	0.404
	Lower content of vitamin C	13	3.4	5	3.9		
	Higher energy value	27	7.1	10	7.9		
	*Higher carbohydrate content*	98	25.7	23	18.1		
	I do not know	234	61.3	83	65.4		

DASH, Dietary approach to stop hypertension; EPA, Eicosapentaenoic acid; DHA, Docosahexaenoic acid; ^†^Chi-square test, ^f^Fisher’s exact test, *statistically significant difference. Correct answers are indicated in italic.

### 3.4 Consumption practices regarding nut consumption

The students’ dietary practices were assessed through 19 questions, eight of which referred to the intake of individual nuts (walnuts, hazelnuts, almonds, cashews, Brazil nuts, pistachios, peanuts, chestnuts).

Walnuts, hazelnuts, almonds, and peanuts are used significantly (above 80%) in students’ regular diets/nutrition, whereas Brazil nuts and chestnuts are the least commonly used. The use of cashews and pistachios is above 40%, which is most likely caused by the price of these products.

Although the majority of the respondents stated that they consumed nuts (81.7%), the main reason why they did not consume nuts was the high price (17.3%), followed by their inappropriate taste (4.7%). This question was open-ended, so respondents could add their answers. Several answers were recorded in which the respondents emphasized that they do not have the habit of using nuts in their diet/nutrition. Those respondents who consumed nuts also had the opportunity to state the reasons for including it in their diet/nutrition. The most common reason was that they like the taste (78.2%), but half of the surveyed students also use nuts because of the health benefits that this food has (51.9%). Of the participants who consume nuts, nearly half cited the beneficial nutritional composition as a reason for including them in their diet. This question was also open-ended, so as an additional answer, it appeared several times that using nuts is a good way to avoid “unhealthy snacks.”

As the main reason for choosing a specific type of nut, the respondents chose taste (69.9%) and price (24.2%), followed by compatibility with other dishes (20.0%) and the opinion that the specific nut is healthy (14.3%).

Almost half of the respondents (47.7%) consumed nuts at least 2–4 times a week, whereas 3 out of the 4 students consumed them at least once a week. The form most often chosen for consumption is raw (52.9%) or baked (40.3%), whereas other forms of consumption are represented to a significantly lesser extent. In regard to the method of consumption, nuts are predominantly taken as snacks. For nuts and commercial snacks, two-thirds of the respondents chose nuts. The feeling of satiety when consuming nuts with a meal was confirmed by 62.2% of the respondents, while one quarter stated that there was no difference.

### 3.5 Main sources of information regarding the health benefits of nut consumption

The questionnaire had questions related to the source of information students use to find nuts: (i) what is the main source of information, (ii) whether the information is obtained on one’s own initiative, (iii) what is the importance of bromatology and nutrition for knowledge in this area, and (iv) whether respondents want to receive additional information on this topic. As the main source of information, slightly more than half of the respondents mentioned lectures at the university (51.9%). However, the majority of the students (60.9%) were informed through mass media (TV, radio, press, internet). Additionally, websites of relevant institutions (37.7%), professional journals, and books (24.4%) were recognized as the main sources of information, while the family was seen in several answers. Almost half of the respondents came to the information about the role and importance of nuts in their own initiative (48.55%), a quarter (24.4%) confirmed that they did not come to the information on their own initiative, and the remaining 27.1% did not remember whose initiative it was.

The impact of the expressed desire to receive additional information about nuts on the answers related to attitudes regarding the health effects and nutritional importance of nuts and knowledge about nuts was also assessed. In half of the statements (statements provided in Table 2), there was a statistically significant difference in attitudes regarding the health effects and nutritional importance of nuts compared with knowledge related to nuts, where a statistically significant difference was seen in only 3 out of 13 questions (questions provided in Table 3). All additional results are provided in the Supplementary material.

## 4 Discussion

To the best of our knowledge, this is the first study to examine attitudes, knowledge and dietary practices related to nut consumption in a sample of pharmacy and non-pharmacy students, and to allow a comparisons between these groups. Furthermore, this study emphasizes the importance of including nutritional topics in the pharmacy curriculum, as future healthcare professionals play an essential role in primary health care by promoting positive attitudes and encouraging the consumption of nuts.

Data from the literature show that the percentage of adults who reach the recommended daily intake of nuts is low (25, 26), especially in UK, New Zealand and the USA, where only up to 7% of adults meet the guidelines (27–30). Nevertheless, the Eat-Lancet diet emphasizes the need to increase nut consumption to 50 g per day (16). In our study, <2% of participants reported not eating nuts, while 36.3% reported eating 10–30 g and 36.0% 30–60 g as their usual portion size. In addition, slightly fewer than three-quarters of the students reported eating nuts once or 2–4 times per week. With respect to data from neighboring countries, the results of a study conducted in Croatia revealed that only 5% of all respondents reported daily and 11% reported weekly nut consumption (31). This slightly higher and more frequent consumption of nuts in our subjects compared to the available data, may be explained by certain characteristics of our subjects such as demographic and lifestyle characteristics (e.g., education level, nonsmoking status, female sex), as described in the literature (11, 31). The consumption of nuts can also depend on dietary habits. In the health-conscious population, e.g., vegans and vegetarians, a higher consumption of nuts was found than in omnivores (22, 25). However, it is interesting to note that the highest percentage of students reported adhering to the regular diet, while vegan and vegetarian diets were almost not represented among the respondents.

While concern about weight gain is often cited as an obstacle to eating nuts (32–35), which is not empirically proven, this concern was less relevant in our sample, which could be due to the higher level of education of our respondents compared to the general population, the main population studied in the literature (34, 35). The fact that more than half of the students cited the health benefits of eating nuts as a reason for eating them and a slightly lower percentage cited the beneficial nutritional composition as a reason also fits in with this results. Price remains a known barrier globally; studies from Australia, New Zealand, China, and the USA identify affordability as a key factor limiting nut consumption (32, 34, 36, 37). However, only a fifth of our participants reported the high price of nuts as the main barrier to nut consumption, this finding is consistent with research on student populations (38). Nut allergies were only mentioned as an obstacle by around 1% of respondents, which is in line with the reported prevalence of allergies to peanuts and tree nuts of between 1 and 4% (39, 40).

Peanuts, walnuts, hazelnuts and almonds were the most commonly consumed, probably due to their affordability and local availability in Serbia. Taste was the most important factor in the choice of nuts, followed by price, which is consistent with studies on consumer preferences (33). In addition to price and taste, one of the reasons for choosing walnuts and hazelnuts as preferred nuts could be that they are grown in the region and are very often part of traditional sweets (41). Similar to study conducted among students in Jordan, our respondents also preferred nuts as snacks, which were mostly consumed raw or roasted (38).

Our study also provides insights into students’ knowledge and perceptions, which are crucial for promoting nut consumption. While the characteristics and barriers associated with nut consumption have been repeatedly highlighted in the literature, few studies have addressed the perceptions and knowledge that can provide additional data for developing strategies to increase nut consumption. In fact, few papers analyzing beliefs, attitudes, barriers, and dietary habits related to nut consumption have been published, while only one study has been conducted with university students (38). In our work, we found that the majority of our participants believe (agree or strongly agree) that nuts are nutrient-rich foods, good sources of protein and unsaturated (good) fats that have a positive impact on health. In addition, more than half of the students believed that eating nuts can help maintain a normal lipid status and that regular and moderate consumption can reduce the risk of cardiovascular disease. In contrast to our findings, more than half of the participants in a study conducted in the USA on attitudes and knowledge in low socioeconomic status populations and individuals diagnosed with cardiovascular disease and/or type 2 diabetes either did not know that eating nuts helps lower cholesterol or did not agree with this statement (34, 35). In a study examining the knowledge, beliefs and attitudes among health professionals (dietitians, general practitioners and nurses), approximately 10% of general practitioners and nurses incorrectly believed that eating nuts can increase the risk of cardiovascular disease, despite epidemiological data consistently showing an inverse correlation between nut consumption and cardiovascular disease risk (21). However, in our study, only 5.1% of the respondents disagreed (strongly disagree or disagree) that nuts reduce the risk of cardiovascular disease.

Furthermore, in the aforementioned studies (33–35), individuals regularly consumed nuts if they were recommended by a doctor. Our results are in agreement with these results, in which half of the respondents stated that they would make an effort to consume nuts more often if they were recommended by a doctor, nutritionist or other health professional.

Nevertheless, in the abovementioned study, which was conducted among different profiles of health professionals (dietitians, GPs and/or practice nurses) (22), as well as a study in which attitudes and knowledge were compared between the general population and health professionals (36), it was concluded that everyone is familiar with the concept of nuts as a healthy food, whereas the general population and health professionals (except dietitians) were not familiar with the specific health benefits associated with the intake of nuts, such as effects on blood cholesterol, CVD risk, and body weight. These results unequivocally indicate that the role of health professionals in promoting the consumption of nuts must be considered in accordance with the requirements to increase the intake of nuts. Furthermore, there is potential for improving knowledge about the benefits of nut consumption among health professionals (especially those who do not specialize in nutrition), which could certainly be part of a strategy to achieve the recommended intake of nuts.

In this context, this work also examines the differences in attitudes and knowledge between students who have subjects related to food or nutrition in their curriculum (food chemistry or nutrition) and the other students included in this study. In fact, the largest number of respondents came from one of the pharmacy schools in Serbia, but only half had taken and passed one or both of the nutrition subjects that are part of the curriculum.

Pharmacists play an important role in primary public health care, not only as experts in medicines but also as the health professionals most accessible to the population (42). As a result, pharmacists are playing an increasingly important role in inpatient counseling for chronic diseases such as obesity, diabetes and hypertension. In addition, one-third of the products sold in pharmacies are nutritional products (e.g., foods for special groups or supplements), so pharmacists need to be knowledgeable about nutrition (42, 43).

However, the literature shows that most pharmacy schools do not have nutrition as a compulsory subject in their curriculum, which is why many pharmacists stated that they are not sufficiently trained in the field of nutrition and do not have sufficient knowledge in this area (44, 45). In contrast to the aforementioned reports, courses on nutrition are mandatory in the curricula of pharmacy schools in Serbia. As our respondents had higher levels of knowledge, beliefs and attitudes than did the literature data did, it was important to investigate the impact of studying these subjects at pharmacy schools compared with other curricula.

When the influence of the faculty that the student attends and the exam previously passed on the statement that respondents know about the health effects of nuts, more than half of the affirmative answers came from students who passed at least one exam on nutrition. In the previously mentioned study comparing the knowledge, attitudes and beliefs of dietitians, general practitioners and nurses (21), it was found that health professionals’ own perceptions of nuts influenced the extent to which they recommended nut consumption to their patients and that differences in health professionals’ levels of knowledge about the benefits of nut consumption influenced their practice of recommending nut consumption to patients. Taken together, these results suggest that pharmacy students’ perceptions as future health professionals with important roles in primary health care are appropriate, which is one of the prerequisites for recommending nut consumption in their professional practice.

In addition, the analysis of the number of correct answers in the total sample or in the sample of students who had passed at least one exam revealed the influence of passing the exam on the score of correct/incorrect/unknown answers, i.e., the group that had passed at least one exam had a significantly lower percentage of scores with 0 correct answers. In this subgroup of students, no participant had 12 or 13 incorrect answers, meaning that every student knew the answers to at least two questions. Interestingly, the respondents in this subgroup gave a lower number of “I do not know” answers. On the basis of these results, we can conclude that despite having passed the exam, pharmacy students still have gaps in their knowledge regarding the nutritional properties and health effects of nuts. In addition, almost a quarter of these students indicated that they did not receive sufficient knowledge about the health importance of nuts in the courses they attended. In addition, ¾ of the students indicated that they would like to receive additional information about the importance of eating nuts, while half of them came to this information on their own initiative. These results can be used to address the importance and role of nuts in the curricula of pharmacy schools, especially as part of a healthy and sustainable diet, which is also a priority in terms of transforming the food system toward sustainability.

By analyzing the impact of attending the pharmacy faculty and passing at least one exam on the topic of food on knowledge about the nutritional characteristics and health effects of nuts, it was determined that a greater impact of passing the exam was manifested in all but one knowledge question, in contrast to the impact of attending the pharmacy faculty, which additionally confirms the previously stated fact about the importance of the existence of subjects on nutrition in the curriculum of pharmacy faculties. However, while knowledge about the consumption of nuts is significantly influenced by passing certain nutrition-related tests, attitudes and beliefs seem to be less influenced by passing the test. Only two attitudes or beliefs showed a statistically significant influence of passing the exam, while attending faculty of pharmacy influenced almost half of the attitudes and beliefs measured. This suggests that the curriculum structure within pharmacy school may play a more important role in shaping general perceptions and attitudes toward nutrition than passing an exam alone.

## 5 Limitations

This study has certain limitations. Firstly, as the questionnaire is intended for self-completion, it is possible that some respondents may not interpret all the questions correctly. To avoid this, the questionnaire was carefully prepared and pretested to identify and eliminate possible ambiguities. The pretest phase focused on face and content validity, while criterion and construct validation was not part of the scope. Second, as the survey was voluntary, there is a possibility that it was only completed by those who were interested in the topic. Third, the majority of respondents were female, which could limit the generalizability of the results to all pharmacy students, although in general, more than 80% of pharmacy students in Serbia are female.

## 6 Conclusion

This study represents a pioneering effort to examine attitudes, knowledge, and dietary practices related to nut consumption in a sample of pharmacy and non-pharmacy students, thereby allowing for the first comparisons between these groups. Our findings emphasize a generally positive perception of nuts and their health benefits among the student population, with consumption rates exceeding those reported in other studies. The results showed that pharmacy students had better knowledge and more positive attitudes toward the consumption of nuts compared to non-pharmacy students. Although completion of nutrition-related courses showed some positive influence, this was not statistically significant for most attitudes and beliefs. These findings underscore the potential value of integrating comprehensive nutrition education into pharmacy curricula, as the combination of knowledge and positive attitudes fostered by pharmacy and nutrition education will enable future health professionals to play a critical role in promoting healthier and sustainable eating habits in the population. Future research should strive to include a more balanced demographic sample, especially by increasing the representation of male students, to ensure the generalizability of the findings.

## Data Availability

The raw data supporting the conclusions of this article will be made available by the authors, without undue reservation.
