# Supplementary material for: The role of nutrition education in the pharmacy curriculum using the example of knowledge about the health benefits of nuts
Source: Front Public Health. 2024 Nov 25;12:1481265. doi: 10.3389/fpubh.2024.1481265 (PMC11625810; doi:10.3389/fpubh.2024.1481265)
Supplement: Supplementary file 1 [file Data_Sheet_1.pdf]

# Supplementary file

***Demographic data (age, height, weight, BMI)***

| | **N** | **Min** | **Max** | **Mean** | **SD** |
|---|---|---|---|---|---|
| **Age (years)** | 509 | 18 | 35 | 23.05 | 2.545 |
| **Height (cm)** | 507 | 150.0 | 200.0 | 171.212 | 8.3437 |
| **Weight (kg)** | 509 | 42.0 | 148.0 | 64.112 | 12.1101 |
| **BMI** $\frac{\boldsymbol{Weight\ (kg)}}{\boldsymbol{(height\ in\ metre)\hat{}2}}$ | 507 | 15.9 | 43.7 | 21.7706 | 2.98811 |

*SD – standard deviation; BMI – body mass index

***Respondents by BMI categories***

| BMI | | **N = 507** | **%** |
|---|---|---|---|
| BMI < 18.5 | **Underweight** | 40 | 7.9 |
| 18.5 ≤ BMI < 25 | **Healthy weight** | 408 | 80.5 |
| 25 ≤ BMI < 30 | **Overweight** | 53 | 10.5 |
| 30 ≤ BMI < 40 | **Obesity** | 4 | 0.8 |
| BMI ≥ 40 | **Extreme obesity** | 2 | 0.4 |

***Information about passed exams in Bromatology and Nutrition***

| | Bromatology (N=509) | | Nutrition (N=509) | |
|---|---|---|---|---|
| | N | % | N | % |
| Yes | 242 | 47.5 | 187 | 36.7 |
| No | 136 | 26.7 | 181 | 35.4 |
| I do not have that exam in my curricula | 131 | 25.7 | 140 | 27.4 |

***Assessment of lifestyle related to physical activity (1 – predominantly sedentary, 5 – very active)***

***Number of hours spent in moderate physical activities and sitting***

| | **N** | **Min** | **Max** | **Mean** | **SD** |
|---|---|---|---|---|---|
| **physical activities (h/week)** | 509 | 0 | 234 | 4.86 | 12.060 |
| **Sitting/lying down (h/day)** | 500 | 1 | 23 | 8.77 | 3.941 |

***List of foods that the respondents marked as allergens***

| | N | % |
|---|---|---|
| Milk and milk products | 9 | 26.47 |
| Eggs | 2 | 5.88 |
| Fish and seafood | 3 | 8.82 |
| Cereals containing gluten (wheat, barley, oats, rye) | 4 | 11.76 |
| Nuts (hazelnuts, almonds, walnuts, cashews, Brazil nuts, pistachios) | 4 | 11.76 |
| Peanuts | 4 | 11.76 |
| Sulfur-dioxide | 1 | 2.94 |
| Orange | 1 | 2.94 |
| Honey | 1 | 2.94 |
| Strawberries | 2 | 5.88 |
| Cherries | 1 | 2.94 |
| Kiwi | 5 | 14.71 |
| Raspberries | 1 | 2.94 |
| Cinnamon | 1 | 2.94 |
| Onion | 1 | 2.94 |
| Apricots | 1 | 2.94 |
| Apples | 2 | 5.88 |

| | | |
|---|---|---|
| Peaches | 2 | 5.88 |

Possession of knowledge about the health effects of nuts

| | | Possession of knowledge about the health effects of nuts | | | |
|---|---|---|---|---|---|
| | | Yes | No | I do not know | Total |
| Faculty type N=509 | Faculty of pharmacy | 279 72.8% out of 383 | 26 | 77 | 383 |
| | Non-pharmacy faculty | 64 50.4% out of 127 | 20 | 43 | 127 |
| | Total | 343 | 46 | 120 | 509 |
| Exam in Bromatology N=509 | Passed | 198 | 7 | 37 | 242 |
| | Not passed | 83 | 15 | 38 | 136 |
| | Do not have exam | 62 47.3% out of 131 | 24 | 45 | 131 |
| | Total | 343 | 46 | 120 | 509 |
| Exam in Nutrition N=508 | Passed | 153 | 6 | 28 | 187 |
| | Not passed | 121 | 15 | 45 | 181 |
| | Do not have exam | 68 48.5% out of 140 | 25 | 47 | 140 |
| | Total | 342 | 46 | 120 | 508 |

***Students' knowledge of nuts***

| **Question** | **Answer** | **Correct answers (N = 509)** | **Correct answers (%)** |
|---|---|---|---|
| **1. In the dietary recommendations (nutrition pyramid), nuts are classified together with the group of foods:** | Cereals | 141 | 27.7 |
| | milk and milk products | 14 | 2.8 |
| | Fruits | 107 | 21.0 |
| | vegetables | 4 | .8 |
| | *meat and eggs* | 67 | 13.2 |
| | Oils | 42 | 8.3 |
| | I do not know | 134 | 26.3 |
| **2. Nuts are an important food group in the Mediterranean diet:** | *Yes* | 300 | 58.9 |
| | No | 35 | 6.9 |
| | I do not know | 174 | 34.2 |
| **3. Nuts are an important food group in the DASH diet:** | *Yes* | 150 | 29.5 |
| | No | 18 | 3.5 |
| | I do not know | 341 | 67.0 |
| **4. Nuts are definitely not a source of which mineral in the diet?** | Selenium | 10 | 2.0 |
| | *Iodine* | 181 | 35.6 |
| | magnesium | 31 | 6.1 |
| | Iron | 31 | 6.1 |
| | Zinc | 30 | 5.9 |
| | I do not know | 226 | 44.4 |
| **5. Nuts are a rich source of which vitamin in the diet?** | vitamin A | 24 | 4.7 |
| | vitamin C | 14 | 2.8 |
| | *vitamin E* | 200 | 39.3 |
| | vitamin D | 19 | 3.7 |
| | vitamin B12 | 88 | 17.3 |

| | | | |
|---|---|---|---|
| | I do not know | 164 | 32.2 |
| **6. Which of the nuts representatives is considered a good source of dietary selenium?** | Almond | 52 | 10.2 |
| | Hazelnut | 8 | 1.6 |
| | *Brazilian nut* | 121 | 23.8 |
| | Walnut | 26 | 5.1 |
| | Cashew | 69 | 13.6 |
| | I do not know | 233 | 45.8 |
| **7. Nuts are primarily a source of which polyunsaturated fatty acid?** | EPA | 39 | 7.7 |
| | DHA | 28 | 5.5 |
| | *linoleic acid* | 42 | 8.3 |
| | alpha-linolenic acid | 63 | 12.4 |
| | arachidonic acid | 35 | 6.9 |
| | I do not know | 302 | 59.3 |
| **8. Which of nuts has the highest content of saturated fatty acids and is therefore the least recommended in the diet compared to other stone fruits?** | Peanuts | 219 | 43.0 |
| | Almond | 30 | 5.9 |
| | Walnut | 23 | 4.5 |
| | Hazelnut | 8 | 1.6 |
| | *Cashew* | 56 | 11.0 |
| | I do not know | 173 | 34.0 |
| **9. There is an approved health claim for walnuts that reads: "Walnuts contribute to improving the elasticity of blood vessels." The stated health statement refers to the daily intake of walnuts in which of the stated quantities?** | 10g | 42 | 8.3 |
| | 15g | 76 | 14.9 |
| | *30g* | 112 | 22.0 |
| | 50g | 37 | 7.3 |
| | 100g | 11 | 2.2 |
| | more than 100g | 1 | .2 |
| | I do not know | 230 | 45.2 |
| **10. The usual thermal processing of nuts has an effect on their chemical composition and thus on their health effects, in which direction?:** | *Lowers* | 261 | 51.3 |
| | increases | 19 | 3.7 |
| | do not change | 64 | 12.6 |
| | I do not know | 165 | 32.4 |

| | | | |
|---|---|---|---|
| **11. Thermal processing of nuts impacts allergenicity, in which direction:** | Lowers | 96 | 18.9 |
| | increases | 39 | 7.7 |
| | *do not change* | 143 | 28.1 |
| | I do not know | 231 | 45.4 |
| **12. According to its composition and health effects, peanuts can be counted as a nut, even though it belongs to the leguminous family:** | *True* | 231 | 45.4 |
| | False | 40 | 7.9 |
| | I do not know | 238 | 46.8 |
| **13. By what characteristic does the chestnut significantly differ from other types of nuts?** | low content of mineral substances | 16 | 3.1 |
| | lower content of vitamin C | 18 | 3.5 |
| | higher energy value | 37 | 7.3 |
| | *higher carbohydrate content* | 121 | 23.8 |
| | I do not know | 317 | 62.3 |

Correct answers are indicated in *italic*.

***Influence of passed exam (Bromatology and/or Nutrition) on Students'knowledge of nuts***

| **Question** | **Answer** | **N = 249** | **%** |
|---|---|---|---|
| **1. In the dietary recommendations (nutrition pyramid), nuts are classified together with the group of foods:** | cereals | 76 | 30.5 |
| | milk and milk products | 10 | 4.0 |
| | Fruits | 71 | 28.5 |
| | vegetables | 4 | 1.6 |
| | *meat and eggs* | 37 | 14.9 |
| | Oils | 22 | 8.8 |
| | I do not know | 29 | 11.6 |
| **2. Nuts are an important food group in the Mediterranean diet:** | *Yes* | 178 | 71.5 |
| | No | 24 | 9.6 |
| | I do not know | 47 | 18.9 |
| **3. Nuts are an important food group in the DASH diet:** | *yes* | 127 | 51.0 |
| | no | 18 | 7.2 |
| | I do not know | 104 | 41.8 |
| **4. Nuts are definitely not a source of which mineral in the diet?** | selenium | 8 | 3.2 |
| | *iodine* | 105 | 42.2 |
| | magnesium | 21 | 8.4 |

| | | | |
|---|---|---|---|
| | iron | 16 | 6.4 |
| | zinc | 16 | 6.4 |
| | I do not know | 83 | 33.3 |
| **5. Nuts are a rich source of which vitamin in the diet?** | vitamin A | 13 | 5.2 |
| | vitamin C | 9 | 3.6 |
| | *vitamin E* | 141 | 56.6 |
| | vitamin D | 8 | 3.2 |
| | vitamin B12 | 36 | 14.5 |
| | I do not know | 42 | 16.9 |
| **6. Which of the nuts representatives is considered a good source of dietary selenium?** | almond | 31 | 12.4 |
| | hazelnut | 5 | 2.0 |
| | *Brazilian nut* | 75 | 30.1 |
| | walnut | 19 | 7.6 |
| | cashew | 40 | 16.1 |
| | I do not know | 79 | 31.7 |
| **7. Nuts are primarily a source of which polyunsaturated fatty acid?** | EPA | 35 | 14.1 |
| | DHA | 23 | 9.2 |
| | *linoleic acid* | 22 | 8.8 |
| | alpha-linolenic acid | 48 | 19.3 |
| | arachidonic acid | 15 | 6.0 |
| | I do not know | 106 | 42.6 |
| **8. Which of nuts has the highest content of saturated fatty acids and is therefore the least recommended in the diet compared to other stone fruits?** | peanuts | 126 | 50.6 |
| | almond | 14 | 5.6 |
| | walnut | 17 | 6.8 |
| | hazelnut | 3 | 1.2 |
| | *cashew* | 32 | 12.9 |
| | I do not know | 57 | 22.9 |
| **9. There is an approved health claim for walnuts that reads: "Walnuts contribute to improving the elasticity of blood vessels." The stated health statement refers to the daily intake of walnuts in which of the** | 10g | 25 | 10.0 |
| | 15g | 37 | 14.9 |
| | *30g* | 66 | 26.5 |

| | | | |
|---|---|---|---|
| **stated quantities?** | 50g | 16 | 6.4 |
| | 100g | 6 | 2.4 |
| | more than 100g | 0 | .0 |
| | I do not know | 99 | 39.8 |
| **10. The usual thermal processing of nuts has an effect on their chemical composition and thus on their health effects, in which direction::** | *lowers* | 147 | 59.0 |
| | increases | 11 | 4.4 |
| | do not change | 34 | 13.7 |
| | I do not know | 57 | 22.9 |
| **11. Thermal processing of nuts impacts allergenicity, in which direction:** | lowers | 59 | 23.7 |
| | increases | 21 | 8.4 |
| | *do not change* | 83 | 33.3 |
| | I do not know | 86 | 34.5 |
| **12. According to its composition and health effects, peanuts can be counted as a nut, even though it belongs to the leguminous family:** | *true* | 129 | 51.8 |
| | false | 24 | 9.6 |
| | I do not know | 96 | 38.6 |
| **13. By what characteristic does the chestnut significantly differ from other types of nuts?** | low content of mineral substances | 9 | 3.6 |
| | lower content of vitamin C | 13 | 5.2 |
| | higher energy value | 24 | 9.6 |
| | *higher carbohydrate content* | 76 | 30.5 |
| | I do not know | 127 | 51.0 |

*Correct answers are indicated in italic.*

***Descriptive statistics of answers on students' knowledge of nuts***

| | | Mean | SD | Median | Min | Max |
|---|---|---|---|---|---|---|
| All respondents (N=509) | Correct | 3.90 | 2.455 | 4 | 0 | 11 |
| | False | 3.35 | 2.176 | 3 | 0 | 11 |
| | Do not know | 5.75 | 3.544 | 5 | 0 | 13 |
| Passed Bromatology and/or Nutrition (N=249) | Correct | 4.89 | 2.356 | 5 | 0 | 11 |
| | False | 4.04 | 2.031 | 4 | 0 | 11 |
| | Do not know | 4.06 | 2.852 | 4 | 0 | 13 |

***Frequency of occurrence of a correct/false response per question***

| | **All respondents** | | | | | | **Passed Bromatology and Nutrition** | | | | | |
|---|---|---|---|---|---|---|---|---|---|---|---|---|
| **Count of answers** | **Correct** | | **False** | | **I do not know** | | **Correct** | | **False** | | **I do not know** | |
| **0** | 47 | 9.2% | 49 | 9.6% | 25 | 4.9% | 5 | 2.0% | 5 | 2.0% | 24 | 9.6% |
| **1** | 42 | 8.3% | 67 | 13.2% | 32 | 6.3% | 10 | 4.0% | 23 | 9.2% | 29 | 11.6% |
| **2** | 69 | 13.6% | 72 | 14.1% | 41 | 8.1% | 21 | 8.4% | 27 | 10.8% | 26 | 10.4% |
| **3** | 70 | 13.8% | 90 | 17.7% | 63 | 12.4% | 37 | 14.9% | 45 | 18.1% | 44 | 17.7% |
| **4** | 89 | 17.5% | 92 | 18.1% | 49 | 9.6% | 45 | 18.1% | 60 | 24.1% | 27 | 10.8% |
| **5** | 66 | 13.0% | 51 | 10.0% | 58 | 11.4% | 40 | 16.1% | 28 | 11.2% | 27 | 10.8% |
| **6** | 48 | 9.4% | 45 | 8.8% | 41 | 8.1% | 29 | 11.6% | 32 | 12.9% | 22 | 8.8% |
| **7** | 37 | 7.3% | 27 | 5.3% | 42 | 8.3% | 27 | 10.8% | 18 | 7.2% | 18 | 7.2% |
| **8** | 22 | 4.3% | 7 | 1.4% | 40 | 7.9% | 17 | 6.8% | 5 | 2.0% | 9 | 3.6% |
| **9** | 8 | 1.6% | 6 | 1.2% | 30 | 5.9% | 8 | 3.2% | 4 | 1.6% | 10 | 4.0% |
| **10** | 7 | 1.4% | 2 | 0.4% | 29 | 5.7% | 6 | 2.4% | 1 | 0.4% | 10 | 4.0% |
| **11** | 4 | 0.8% | 1 | 0.2% | 15 | 2.9% | 4 | 1.6% | 1 | 0.4% | 1 | 0.4% |
| **12** | 0 | 0.0% | 0 | 0.0% | 18 | 3.5% | 0 | 0.0% | 0 | 0.0% | 1 | 0.4% |
| **13** | 0 | 0.0% | 0 | 0.0% | 26 | 5.1% | 0 | 0.0% | 0 | 0.0% | 1 | 0,4% |

***Reasons why nuts are not consumed by respondents***

| | N = 509 | % |
|---|---|---|
| Nut allergy | 5 | 0.98 |
| Inappropriate taste | 24 | 4.72 |
| I do not see the importance of nuts in the diet | 3 | 0.59 |
| High cost of nuts | 88 | 17.29 |
| Nuts are high in calories and contribute to weight gain | 9 | 1.77 |
| I believe that nuts increase blood cholesterol | 3 | 0.59 |
| I believe that nuts increase the risk of developing cardiovascular disease | 3 | 0.59 |
| I eat nuts | 416 | 81.73 |

***Reasons why nuts are consumed by respondents***

| | N = 509 | % |
|---|---|---|
| Because of the health benefits it has | 264 | 51.87 |
| Because of the good nutritional composition | 233 | 45.78 |
| I love the taste of nuts | 398 | 78.19 |
| I don't consume nuts | 24 | 4.72 |

***Cross-comparison of reasons for consuming nuts with previously passed exams***

| | Bromatology (N = 509) | | | Nutrition (N = 508) | | |
|---|---|---|---|---|---|---|
| | Passed (N = 242) | Not passed (N = 135) | No exam in curricula (N = 132) | Passed (N = 187) | Not passed (N = 181) | No exam in curricula (N = 140) |
| Because of the health benefits it has | 134 | 72 | 58 | 99 | 87 | 77 |
| Because of the good nutritional composition | 115 | 65 | 53 | 95 | 74 | 64 |
| I love the taste of nuts | 192 | 103 | 103 | 149 | 135 | 114 |
| I don't consume nuts | 10 | 4 | 10 | 6 | 12 | 6 |

***The type of nuts that is most common consumed in the diet.***

| | N = 509 | % |
|---|---|---|
| Walnut | 76 | 14.9 |
| Hazelnut | 80 | 15.7 |
| Almond | 154 | 30.3 |
| Cashew | 18 | 3.5 |
| Brazilian nut | 3 | 0.6 |
| Pistachios | 13 | 2.5 |
| Peanuts | 153 | 30.1 |
| Tame chestnut | 3 | 0.6 |
| I don't consume nuts | 9 | 1.8 |

***Frequency of use of nuts in diet***

| | N = 509 | % |
|---|---|---|
| More than 5 times a week | 43 | 8.4 |
| 2-4 times a week | 200 | 39.3 |
| once a week | 142 | 27.9 |
| 3 times a month | 76 | 14.9 |
| Less than once a month | 41 | 8.1 |
| I don't consume nuts | 7 | 1.4 |

***Usual portion size of nuts in the diet***

| | N = 509 | % |
|---|---|---|
| 10-30g | 185 | 36.3 |
| 30-60g | 183 | 36.0 |
| 60-90g | 83 | 16.3 |
| 90-120g | 39 | 7.7 |
| 120-150g | 8 | 1.6 |
| More than 150g | 3 | 0.6 |
| I do not consume nuts | 8 | 1.6 |

***The usual form of nuts consumption***

| | N = 509 | % |
|---|---|---|
| Raw | 269 | 52.9 |
| Baked | 205 | 40.3 |
| Fried in oil | 12 | 2.4 |
| In the form of paste, butter | 13 | 2.6 |
| I do not consume nuts | 9 | 1.8 |
| Other: stayed in water for some time | 1 | 0.2 |

***Type of nuts procurement***

| | N = 509 | % |
|---|---|---|
| Industrially packaged | 101 | 19.8 |
| In bulk goods stores ("bio shop"), by measure | 326 | 64.0 |
| On the market, by measure | 62 | 12.2 |
| I do not consume nuts | 8 | 1.6 |
| Other (independent cultivation, multiple answers) | 12 | 2.4 |

***The association of the need for additional information with attitudes regarding the health effects and nutritional value of nuts***

| 1. Nuts represent a group of foods of exceptional nutritional value. | | | | | | | | |
|---|---|---|---|---|---|---|---|---|
| Need for additional information | | 1 | 2 | 3 | 4 | 5 | Stat. | p-value |
| Yes | N = 380 | 1 | 8 | 42 | 114 | 215 | 33.947 | <0.001* |
| | % | 0.3 | 2.1 | 11.1 | 30.0 | 56.6 | | |
| No | N = 49 | 2 | 3 | 12 | 11 | 21 | | |
| | % | 4.1 | 6.1 | 24.5 | 22.4 | 42.9 | | |
| I do not know | N = 80 | 0 | 3 | 21 | 25 | 31 | | |
| | % | 0.0 | 3.8 | 26.3 | 31.3 | 38.8 | | |
| 2. Regular consumption of nuts can have beneficial effects on health. | | | | | | | | |
| Need for additional information | | 1 | 2 | 3 | 4 | 5 | Stat. | p-value |
| Yes | N = 380 | 0 | 5 | 36 | 112 | 227 | 51.763 | <0.001* |
| | % | 0.0 | 1.3 | 9.5 | 29.5 | 59.7 | | |
| No | N = 49 | 1 | 7 | 13 | 8 | 20 | | |
| | % | 2.0 | 14.3 | 26.5 | 16.3 | 40.8 | | |
| I do not know | N = 80 | 0 | 4 | 13 | 24 | 39 | | |

| | | | | | | | | |
|---|---|---|---|---|---|---|---|---|
| | % | 0.0 | 5.0 | 16.3 | 30.0 | 48.8 | | |
| 3. Consuming nuts contributes to the maintenance of normal lipid status. | | | | | | | | |
| Need for additional information | | 1 | 2 | 3 | 4 | 5 | Stat. | p-value |
| Yes | N = 379 | 2 | 12 | 99 | 115 | 151 | 31.646 | <0.001* |
| | % | .5 | 3.2 | 26.1 | 30.3 | 39.8 | | |
| No | N = 49 | 4 | 4 | 17 | 8 | 16 | | |
| | % | 8.2 | 8.2 | 34.7 | 16.3 | 32.7 | | |
| I do not know | N = 80 | 0 | 5 | 25 | 22 | 28 | | |
| | % | 0.0 | 6.3 | 31.3 | 27.5 | 35.0 | | |
| 4. Regular and moderate consumption of nuts contributes to reducing the risk of developing cardiovascular diseases. | | | | | | | | |
| Need for additional information | | 1 | 2 | 3 | 4 | 5 | Stat. | p-value |
| Yes | N = 380 | 1 | 15 | 84 | 128 | 152 | 18.493 | 0.018* |
| | % | .3 | 3.9 | 22.1 | 33.7 | 40.0 | | |
| No | N = 49 | 2 | 5 | 16 | 11 | 15 | | |
| | % | 4.1 | 10.2 | 32.7 | 22.4 | 30.6 | | |
| I do not know | N = 80 | 1 | 2 | 19 | 30 | 28 | | |
| | % | 1.3 | 2.5 | 23.8 | 37.5 | 35.0 | | |
| 5. Regular and moderate consumption of nuts contributes to reducing the risk of developing diabetes. | | | | | | | | |
| Need for additional information | | 1 | 2 | 3 | 4 | 5 | Stat. | p-value |
| Yes | N = 380 | 5 | 36 | 115 | 115 | 109 | 13.583 | 0.093 |
| | % | 1.3 | 9.5 | 30.3 | 30.3 | 28.7 | | |
| No | N = 49 | 4 | 7 | 15 | 11 | 12 | | |
| | % | 8.2 | 14.3 | 30.6 | 22.4 | 24.5 | | |
| I do not know | N = 80 | 1 | 9 | 25 | 26 | 19 | | |
| | % | 1.3 | 11.3 | 31.3 | 32.5 | 23.8 | | |
| 6. Regular and moderate consumption of nuts contributes to reducing the risk of cancer. | | | | | | | | |

| Need for additional information | | 1 | 2 | 3 | 4 | 5 | Stat. | p-value |
|---|---|---|---|---|---|---|---|---|
| Yes | N = 380 | 10 | 59 | 134 | 96 | 81 | 14.628 | 0.067 |
| | % | 2.6 | 15.5 | 35.3 | 25.3 | 21.3 | | |
| No | N = 49 | 5 | 13 | 17 | 5 | 9 | | |
| | % | 10.2 | 26.5 | 34.7 | 10.2 | 18.4 | | |
| I do not know | N = 80 | 4 | 13 | 28 | 19 | 16 | | |
| | % | 5.0 | 16.3 | 35.0 | 23.8 | 20.0 | | |
| 7. Regular and moderate consumption of nuts contributes to an increase in total cholesterol. | | | | | | | | |
| Need for additional information | | 1 | 2 | 3 | 4 | 5 | Stat. | p-value |
| Yes | N = 380 | 52 | 107 | 121 | 63 | 37 | 5.933 | 0.655 |
| | % | 13.7 | 28.2 | 31.8 | 16.6 | 9.7 | | |
| No | N = 49 | 8 | 13 | 20 | 5 | 3 | | |
| | % | 16.3 | 26.5 | 40.8 | 10.2 | 6.1 | | |
| I do not know | N = 80 | 7 | 20 | 32 | 12 | 9 | | |
| | % | 8.8 | 25.0 | 40.0 | 15.0 | 11.3 | | |
| 8. Consuming nuts several times a week contributes to an increase in body weight. | | | | | | | | |
| Need for additional information | | 1 | 2 | 3 | 4 | 5 | Stat. | p-value |
| Yes | N = 380 | 90 | 95 | 105 | 67 | 23 | 2.932 | 0.939 |
| | % | 23.7 | 25.0 | 27.6 | 17.6 | 6.1 | | |
| No | N = 49 | 11 | 10 | 15 | 9 | 4 | | |
| | % | 22.4 | 20.4 | 30.6 | 18.4 | 8.2 | | |
| I do not know | N = 80 | 19 | 18 | 28 | 11 | 4 | | |
| | % | 23.8 | 22.5 | 35.0 | 13.8 | 5.0 | | |
| 9. Regular consumption of nuts contributes to the reduction of inflammation. | | | | | | | | |
| Need for additional information | | 1 | 2 | 3 | 4 | 5 | Stat. | p-value |
| Yes | N = 379 | 15 | 53 | 182 | 83 | 46 | 11.686 | 0.166 |

| | | | | | | | | |
|---|---|---|---|---|---|---|---|---|
| | % | 4.0 | 14.0 | 48.0 | 21.9 | 12.1 | | |
| No | N = 49 | 5 | 9 | 23 | 6 | 6 | | |
| | % | 10.2 | 18.4 | 46.9 | 12.2 | 12.2 | | |
| I do not know | N = 80 | 4 | 17 | 42 | 12 | 5 | | |
| | % | 5.0 | 21.3 | 52.5 | 15.0 | 6.3 | | |
| 10. Regular consumption of nuts contributes to the maintenance of normal endothelial function. | | | | | | | | |
| Need for additional information | | 1 | 2 | 3 | 4 | 5 | Stat. | p-value |
| Yes | N = 379 | 6 | 42 | 152 | 119 | 60 | 21.531 | 0.006* |
| | % | 1.6 | 11.1 | 40.1 | 31.4 | 15.8 | | |
| No | N = 49 | 4 | 7 | 25 | 7 | 6 | | |
| | % | 8.2 | 14.3 | 51.0 | 14.3 | 12.2 | | |
| I do not know | N = 80 | 2 | 3 | 42 | 25 | 8 | | |
| | % | 2.5 | 3.8 | 52.5 | 31.3 | 10.0 | | |
| 11. Regular consumption of nuts can have a beneficial effect on the oxidative status in the body. | | | | | | | | |
| Need for additional information | | 1 | 2 | 3 | 4 | 5 | Stat. | p-value |
| Yes | N = 379 | 0 | 34 | 108 | 132 | 105 | 50.214 | <0.001* |
| | % | 0.0 | 9.0 | 28.5 | 34.8 | 27.7 | | |
| No | N = 49 | 4 | 6 | 20 | 12 | 7 | | |
| | % | 8.2 | 12.2 | 40.8 | 24.5 | 14.3 | | |
| I do not know | N = 80 | 0 | 4 | 28 | 34 | 14 | | |
| | % | 0.0 | 5.0 | 35.0 | 42.5 | 17.5 | | |
| 12. It is important to know that nuts represent a source of protein in the diet. | | | | | | | | |
| Need for additional information | | 1 | 2 | 3 | 4 | 5 | Stat. | p-value |
| Yes | N = 380 | 22 | 39 | 71 | 108 | 140 | 13.337 | 0.101 |
| | % | 5.8 | 10.3 | 18.7 | 28.4 | 36.8 | | |
| No | N = 49 | 5 | 11 | 10 | 7 | 16 | | |
| | % | 10.2 | 22.4 | 20.4 | 14.3 | 32.7 | | |

| | | | | | | | | |
|---|---|---|---|---|---|---|---|---|
| I do not know | N = 80 | 6 | 7 | 20 | 23 | 24 | | |
| | % | 7.5 | 8.8 | 25.0 | 28.8 | 30.0 | | |
| 13. Nuts should be consumed regularly because it is a good source of unsaturated (healthy) fats in the diet. | | | | | | | | |
| Need for additional information | | 1 | 2 | 3 | 4 | 5 | Stat. | p-value |
| Yes | N = 380 | 1 | 13 | 57 | 102 | 207 | 28.905 | <0.001* |
| | % | 0.3 | 3.4 | 15.0 | 26.8 | 54.5 | | |
| No | N = 49 | 2 | 7 | 12 | 9 | 19 | | |
| | % | 4.1 | 14.3 | 24.5 | 18.4 | 38.8 | | |
| I do not know | N = 80 | 0 | 5 | 16 | 22 | 37 | | |
| | % | 0.0 | 6.3 | 20.0 | 27.5 | 46.3 | | |
| 14. Nuts should be consumed regularly because it is a good source of essential fatty acids in the diet. | | | | | | | | |
| Need for additional information | | 1 | 2 | 3 | 4 | 5 | Stat. | p-value |
| Yes | N = 380 | 4 | 12 | 84 | 105 | 175 | 26.023 | 0.001 |
| | % | 1.1 | 3.2 | 22.1 | 27.6 | 46.1 | | |
| No | N = 49 | 4 | 5 | 14 | 10 | 16 | | |
| | % | 8.2 | 10.2 | 28.6 | 20.4 | 32.7 | | |
| I do not know | N = 80 | 1 | 6 | 21 | 26 | 26 | | |
| | % | 1.3 | 7.5 | 26.3 | 32.5 | 32.5 | | |
| 15. Nuts can contribute to the preservation and improvement of health as a significant source of antioxidants. | | | | | | | | |
| Need for additional information | | 1 | 2 | 3 | 4 | 5 | Stat. | p-value |
| Yes | N = 379 | 4 | 18 | 69 | 131 | 157 | 32.191 | <0.001* |
| | % | 1.1 | 4.7 | 18.2 | 34.6 | 41.4 | | |
| No | N = 49 | 5 | 3 | 16 | 6 | 19 | | |
| | % | 10.2 | 6.1 | 32.7 | 12.2 | 38.8 | | |
| I do not know | N = 80 | 2 | 4 | 22 | 28 | 24 | | |
| | % | 2.5 | 5.0 | 27.5 | 35.0 | 30.0 | | |
| 16. Almonds are a good nutritional source of iron in the diet. | | | | | | | | |

| Need for additional information | | 1 | 2 | 3 | 4 | 5 | Stat. | p-value |
|---|---|---|---|---|---|---|---|---|
| Yes | N = 380 | 19 | 52 | 117 | 103 | 89 | 9.149 | 0.330 |
| | % | 5.0 | 13.7 | 30.8 | 27.1 | 23.4 | | |
| No | N = 49 | 5 | 7 | 17 | 10 | 10 | | |
| | % | 10.2 | 14.3 | 34.7 | 20.4 | 20.4 | | |
| I do not know | N = 80 | 9 | 6 | 29 | 19 | 17 | | |
| | % | 11.3 | 7.5 | 36.3 | 23.8 | 21.3 | | |
| 17. Nuts should be recommended as a healthy snack in the diet. | | | | | | | | |
| Need for additional information | | 1 | 2 | 3 | 4 | 5 | Stat. | p-value |
| Yes | N = 380 | 4 | 8 | 35 | 93 | 240 | 28.024 | <0.001* |
| | % | 1.1 | 2.1 | 9.2 | 24.5 | 63.2 | | |
| No | N = 49 | 2 | 5 | 11 | 11 | 20 | | |
| | % | 4.1 | 10.2 | 22.4 | 22.4 | 40.8 | | |
| I do not know | N = 80 | 1 | 4 | 13 | 25 | 37 | | |
| | % | 1.3 | 5.0 | 16.3 | 31.3 | 46.3 | | |
| 18. The general population is not sufficiently informed about the health benefits of nuts. | | | | | | | | |
| Need for additional information | | 1 | 2 | 3 | 4 | 5 | Stat. | p-value |
| Yes | N = 380 | 3 | 10 | 44 | 76 | 247 | 37.259 | <0.001* |
| | % | 0.8 | 2.6 | 11.6 | 20.0 | 65.0 | | |
| No | N = 49 | 4 | 5 | 11 | 6 | 23 | | |
| | % | 8.2 | 10.2 | 22.4 | 12.2 | 46.9 | | |
| I do not know | N = 80 | 2 | 3 | 17 | 22 | 36 | | |
| | % | 2.5 | 3.8 | 21.3 | 27.5 | 45.0 | | |
| 19. One of the main barriers to the regular consumption of nuts is the high price. | | | | | | | | |
| Need for additional information | | 1 | 2 | 3 | 4 | 5 | Stat. | p-value |
| Yes | N = 380 | 9 | 21 | 60 | 117 | 173 | 20.073 | 0.010* |

| | | | | | | | | |
|---|---|---|---|---|---|---|---|---|
| | % | 2.4 | 5.5 | 15.8 | 30.8 | 45.5 | | |
| No | N = 49 | 4 | 7 | 13 | 9 | 16 | | |
| | % | 8.2 | 14.3 | 26.5 | 18.4 | 32.7 | | |
| I do not know | N = 80 | 0 | 6 | 14 | 24 | 36 | | |
| | % | 0.0 | 7.5 | 17.5 | 30.0 | 45.0 | | |
| 20. I rarely consume nuts for fear of gaining weight. | | | | | | | | |
| Need for additional information | | 1 | 2 | 3 | 4 | 5 | Stat. | p-value |
| Yes | N = 380 | 220 | 58 | 52 | 30 | 20 | 8.128 | 0.421 |
| | % | 57.9 | 15.3 | 13.7 | 7.9 | 5.3 | | |
| No | N = 49 | 28 | 5 | 12 | 2 | 2 | | |
| | % | 57.1 | 10.2 | 24.5 | 4.1 | 4.1 | | |
| I do not know | N = 80 | 45 | 8 | 17 | 7 | 3 | | |
| | % | 56.3 | 10.0 | 21.3 | 8.8 | 3.8 | | |
| 21. I would try to consume nuts more often if recommended by a doctor, nutritionist or other health professional. | | | | | | | | |
| Need for additional information | | 1 | 2 | 3 | 4 | 5 | Stat. | p-value |
| Yes | N = 380 | 44 | 40 | 84 | 82 | 130 | 27.393 | 0.001* |
| | % | 11.6 | 10.5 | 22.1 | 21.6 | 34.2 | | |
| No | N = 49 | 17 | 4 | 12 | 7 | 9 | | |
| | % | 34.7 | 8.2 | 24.5 | 14.3 | 18.4 | | |
| I do not know | N = 80 | 9 | 12 | 25 | 14 | 20 | | |
| | % | 11.3 | 15.0 | 31.3 | 17.5 | 25.0 | | |
| 22. Nuts are one of the most common causes of allergic reactions to food. | | | | | | | | |
| Need for additional information | | 1 | 2 | 3 | 4 | 5 | Stat. | p-value |
| Yes | N = 380 | 20 | 41 | 87 | 121 | 111 | 11.425 | 0.179 |
| | % | 5.3 | 10.8 | 22.9 | 31.8 | 29.2 | | |
| No | N = 49 | 6 | 8 | 14 | 13 | 8 | | |

| | | | | | | | | |
|---|---|---|---|---|---|---|---|---|
| | % | 12.2 | 16.3 | 28.6 | 26.5 | 16.3 | | |
| I do not know | N = 80 | 8 | 7 | 16 | 22 | 27 | | |
| | % | 10.0 | 8.8 | 20.0 | 27.5 | 33.8 | | |

### *The association between the need for additional information and students' knowledge about nuts*

| | | Yes (N=380) | | No (N=49) | | I don't know (N=80) | | Test | |
|---|---|---|---|---|---|---|---|---|---|
| **Question** | **Answer** | **N** | **%** | **N** | **%** | **N** | **%** | **Stat.** | **p** |
| **1. In the dietary recommendations (nutrition pyramid), nuts are classified together with the group of foods:** | Cereals | 105 | 27.6 | 13 | 26.5 | 23 | 28.8 | 19.406 | 0.079 |
| | milk and milk products | 12 | 3.2 | 2 | 4.1 | 0 | 0.0 | | |
| | Fruits | 88 | 23.2 | 5 | 10.2 | 14 | 17.5 | | |
| | Vegetables | 3 | 0.8 | 0 | 0.0 | 1 | 1.3 | | |
| | *meat and eggs* | 55 | 14.5 | 8 | 16.3 | 4 | 5.0 | | |
| | Oils | 26 | 6.8 | 6 | 12.2 | 10 | 12.5 | | |
| | I do not know | 91 | 23.9 | 15 | 30.6 | 28 | 35.0 | | |
| **2. Nuts are an important food group in the Mediterranean diet:** | *Yes* | 238 | 62.6 | 25 | 51.0 | 37 | 46.3 | 10.638 | 0.031 |
| | No | 27 | 7.1 | 3 | 6.1 | 5 | 6.3 | | |
| | I do not know | 115 | 30.3 | 21 | 42.9 | 38 | 47.5 | | |
| **3. Nuts are an important food group in the DASH diet:** | *Yes* | 119 | 31.3 | 11 | 22.4 | 20 | 25.0 | 3.661 | 0.454 |
| | No | 13 | 3.4 | 3 | 6.1 | 2 | 2.5 | | |
| | I do not know | 248 | 65.3 | 35 | 71.4 | 58 | 72.5 | | |
| **4. Nuts are definitely not a source of which mineral in the diet?** | Selenium | 7 | 1.8 | 2 | 4.1 | 1 | 1.3 | 13.059 | 0.220 |
| | *Iodine* | 144 | 37.9 | 13 | 26.5 | 24 | 30.0 | | |
| | Magnesium | 21 | 5.5 | 1 | 2.0 | 9 | 11.3 | | |
| | Iron | 22 | 5.8 | 2 | 4.1 | 7 | 8.8 | | |
| | Zinc | 23 | 6.1 | 2 | 4.1 | 5 | 6.3 | | |

| | | | | | | | | | |
|---|---|---|---|---|---|---|---|---|---|
| | I do not know | 163 | 42.9 | 29 | 59.2 | 34 | 42.5 | | |
| **5. Nuts are a rich source of which vitamin in the diet?** | vitamin A | 18 | 4.7 | 3 | 6.1 | 3 | 3.8 | 11.678 | 0.307 |
| | vitamin C | 7 | 1.8 | 3 | 6.1 | 4 | 5.0 | | |
| | *vitamin E* | 159 | 41.8 | 15 | 30.6 | 26 | 32.5 | | |
| | vitamin D | 12 | 3.2 | 2 | 4.1 | 5 | 6.3 | | |
| | vitamin B12 | 68 | 17.9 | 6 | 12.2 | 14 | 17.5 | | |
| | I do not know | 116 | 30.5 | 20 | 40.8 | 28 | 35.0 | | |
| **6. Which of the nuts representatives is considered a good source of dietary selenium?** | Almond | 37 | 9.7 | 3 | 6.1 | 12 | 15.0 | 9.231 | 0.510 |
| | Hazelnut | 4 | 1.1 | 1 | 2.0 | 3 | 3.8 | | |
| | *Brazilian nut* | 97 | 25.5 | 11 | 22.4 | 13 | 16.3 | | |
| | Walnut | 21 | 5.5 | 2 | 4.1 | 3 | 3.8 | | |
| | Cashew | 51 | 13.4 | 7 | 14.3 | 11 | 13.8 | | |
| | I do not know | 170 | 44.7 | 25 | 51.0 | 38 | 47.5 | | |
| **7. Nuts are primarily a source of which polyunsaturated fatty acid?** | EPA | 25 | 6.6 | 5 | 10.2 | 9 | 11.3 | 7.573 | 0.670 |
| | DHA | 24 | 6.3 | 2 | 4.1 | 2 | 2.5 | | |
| | *linoleic acid* | 34 | 8.9 | 3 | 6.1 | 5 | 6.3 | | |
| | alpha-linolenic acid | 48 | 12.6 | 7 | 14.3 | 8 | 10.0 | | |
| | arachidonic acid | 29 | 7.6 | 2 | 4.1 | 4 | 5.0 | | |
| | I do not know | 220 | 57.9 | 30 | 61.2 | 52 | 65.0 | | |
| **8. Which of nuts has the highest content of saturated fatty acids and is therefore the least recommended in the diet compared to other stone fruits?** | Peanuts | 172 | 45.3 | 15 | 30.6 | 32 | 40.0 | 14.762 | 0.141 |
| | Almond | 20 | 5.3 | 2 | 4.1 | 8 | 10.0 | | |
| | Walnut | 14 | 3.7 | 4 | 8.2 | 5 | 6.3 | | |
| | Hazelnut | 7 | 1.8 | 0 | 0.0 | 1 | 1.3 | | |
| | *Cashew* | 43 | 11.3 | 3 | 6.1 | 10 | 12.5 | | |
| | I do not know | 124 | 32.6 | 25 | 51.0 | 24 | 30.0 | | |
| **9. There is an approved health claim for walnuts that reads: "Walnuts contribute to improving the elasticity of blood vessels." The stated** | 10g | 33 | 8.7 | 4 | 8.2 | 5 | 6.3 | 4.160 | 0.980 |
| | 15g | 58 | 15.3 | 7 | 14.3 | 11 | 13.8 | | |
| | *30g* | 85 | 22.4 | 11 | 22.4 | 16 | 20.0 | | |
| | 50g | 30 | 7.9 | 3 | 6.1 | 4 | 5.0 | | |
| | 100g | 7 | 1.8 | 2 | 4.1 | 2 | 2.5 | | |

| | | | | | | | | | |
|---|---|---|---|---|---|---|---|---|---|
| **health statement refers to the daily intake of walnuts in which of the stated quantities?** | more than 100g | 1 | 0.3 | 0 | 0.0 | 0 | 0.0 | | |
| | I do not know | 166 | 43.7 | 22 | 44.9 | 42 | 52.5 | | |
| **10. The usual thermal processing of nuts has an effect on their chemical composition and thus on their health effects, in which direction: :** | *Lowers* | 209 | 55.0 | 17 | 34.7 | 35 | 43.8 | 11.891 | 0.064 |
| | increases | 13 | 3.4 | 4 | 8.2 | 2 | 2.5 | | |
| | do not change | 46 | 12.1 | 7 | 14.3 | 11 | 13.8 | | |
| | I do not know | 112 | 29.5 | 21 | 42.9 | 32 | 40.0 | | |
| **11. Thermal processing of nuts impacts allergenicity, in which direction:** | Lowers | 69 | 18.2 | 9 | 18.4 | 18 | 22.5 | 2.560 | 0.862 |
| | increases | 31 | 8.2 | 3 | 6.1 | 5 | 6.3 | | |
| | *do not change* | 109 | 28.7 | 11 | 22.4 | 23 | 28.8 | | |
| | I do not know | 171 | 45.0 | 26 | 53.1 | 34 | 42.5 | | |
| **12. According to its composition and health effects, peanuts can be counted as a nut, even though it belongs to the leguminous family:** | *True* | 180 | 47.4 | 16 | 32.7 | 35 | 43.8 | 6.422 | 0.170 |
| | False | 25 | 6.6 | 7 | 14.3 | 8 | 10.0 | | |
| | I do not know | 175 | 46.1 | 26 | 53.1 | 37 | 46.3 | | |
| **13. By what characteristic does the chestnut significantly differ from other types of nuts?** | low content of mineral substances | 13 | 3.4 | 1 | 2.0 | 2 | 2.5 | 4.198 | 0.839 |
| | lower content of vitamin C | 11 | 2.9 | 3 | 6.1 | 4 | 5.0 | | |
| | higher energy value | 26 | 6.8 | 4 | 8.2 | 7 | 8.8 | | |
| | *higher carbohydrate content* | 96 | 25.3 | 9 | 18.4 | 16 | 20.0 | | |
| | I do not know | 234 | 61.6 | 32 | 65.3 | 51 | 63.8 | | |

***Correct answers are indicated in italic.***

***The influence of passing the nutrition-related exam (Bromatology) on attitudes regarding the health effects and nutritional importance of nuts***

| | | | | | | | | |
|---|---|---|---|---|---|---|---|---|
| 1. Nuts represent a group of foods of exceptional nutritional value. | | | | | | | | |
| | | 1 | 2 | 3 | 4 | 5 | Stat. | p-value |
| Passed | N = 242 | 1 | 5 | 34 | 69 | 133 | 8.330[f] | 0.366 |
| | % | .4 | 2.1 | 14.0 | 28.5 | 55.0 | | |
| Not passed | N = 136 | 1 | 5 | 16 | 49 | 65 | | |
| | % | .7 | 3.7 | 11.8 | 36.0 | 47.8 | | |
| No exam in curricula | N = 131 | 1 | 4 | 25 | 32 | 69 | | |
| | % | .8 | 3.1 | 19.1 | 24.4 | 52.7 | | |
| 2. Regular consumption of nuts can have beneficial effects on health. | | | | | | | | |
| | | 1 | 2 | 3 | 4 | 5 | Stat. | p-value |
| Passed | N =242 | 0 | 4 | 18 | 68 | 152 | 22.914[f] | 0.001* |
| | % | 0.0 | 1.7 | 7.4 | 28.1 | 62.8 | | |
| Not passed | N =136 | 0 | 4 | 19 | 37 | 76 | | |
| | % | 0.0 | 2.9 | 14.0 | 27.2 | 55.9 | | |
| No exam in curricula | N =131 | 1 | 8 | 25 | 39 | 58 | | |
| | % | .8 | 6.1 | 19.1 | 29.8 | 44.3 | | |
| 3. Consuming nuts contributes to the maintenance of normal lipid status. | | | | | | | | |
| | | 1 | 2 | 3 | 4 | 5 | Stat. | p-value |
| Passed | N =242 | 1 | 6 | 54 | 78 | 103 | 20.182 | 0.010* |
| | % | .4 | 2.5 | 22.3 | 32.2 | 42.6 | | |
| Not passed | N =135 | 3 | 7 | 36 | 38 | 51 | | |
| | % | 2.2 | 5.2 | 26.7 | 28.1 | 37.8 | | |
| No exam in curricula | N =131 | 2 | 8 | 51 | 29 | 41 | | |
| | % | 1.5 | 6.1 | 38.9 | 22.1 | 31.3 | | |
| 4. Regular and moderate consumption of nuts contributes to reducing the risk of developing cardiovascular diseases. | | | | | | | | |
| | | 1 | 2 | 3 | 4 | 5 | Stat. | p-value |
| Passed | N =242 | 0 | 9 | 48 | 85 | 100 | 18.965 | 0.015* |
| | % | 0.0 | 3.7 | 19.8 | 35.1 | 41.3 | | |
| Not passed | N =136 | 0 | 5 | 34 | 42 | 55 | | |

| | | | | | | | | |
|---|---|---|---|---|---|---|---|---|
| | % | 0.0 | 3.7 | 25.0 | 30.9 | 40.4 | | |
| No exam in curricula | N =131 | 4 | 8 | 37 | 42 | 40 | | |
| | % | 3.1 | 6.1 | 28.2 | 32.1 | 30.5 | | |
| 5. Regular and moderate consumption of nuts contributes to reducing the risk of developing diabetes. | | | | | | | | |
| | | 1 | 2 | 3 | 4 | 5 | Stat. | p-value |
| Passed | N =242 | 3 | 24 | 67 | 78 | 70 | 8.368 | 0.398 |
| | % | 1.2 | 9.9 | 27.7 | 32.2 | 28.9 | | |
| Not passed | N =136 | 3 | 13 | 38 | 42 | 40 | | |
| | % | 2.2 | 9.6 | 27.9 | 30.9 | 29.4 | | |
| No exam in curricula | N =131 | 4 | 15 | 50 | 32 | 30 | | |
| | % | 3.1 | 11.5 | 38.2 | 24.4 | 22.9 | | |
| 6. Regular and moderate consumption of nuts contributes to reducing the risk of cancer. | | | | | | | | |
| | | 1 | 2 | 3 | 4 | 5 | Stat. | p-value |
| Passed | N =242 | 9 | 43 | 83 | 59 | 48 | 10.485 | 0.233 |
| | % | 3.7 | 17.8 | 34.3 | 24.4 | 19.8 | | |
| Not passed | N =136 | 3 | 16 | 47 | 32 | 38 | | |
| | % | 2.2 | 11.8 | 34.6 | 23.5 | 27.9 | | |
| No exam in curricula | N =131 | 7 | 26 | 49 | 29 | 20 | | |
| | % | 5.3 | 19.8 | 37.4 | 22.1 | 15.3 | | |
| 7. Regular and moderate consumption of nuts contributes to an increase in total cholesterol. | | | | | | | | |
| | | 1 | 2 | 3 | 4 | 5 | Stat. | p-value |
| Passed | N =242 | 35 | 72 | 73 | 39 | 23 | 4.729 | 0.786 |
| | % | 14.5 | 29.8 | 30.2 | 16.1 | 9.5 | | |
| Not passed | N =136 | 14 | 33 | 54 | 21 | 14 | | |
| | % | 10.3 | 24.3 | 39.7 | 15.4 | 10.3 | | |
| No exam in curricula | N =131 | 18 | 35 | 46 | 20 | 12 | | |
| | % | 13.7 | 26.7 | 35.1 | 15.3 | 9.2 | | |
| 8. Consuming nuts several times a week contributes to an increase in body weight. | | | | | | | | |
| | | 1 | 2 | 3 | 4 | 5 | Stat. | p-value |
| Passed | N =242 | 64 | 56 | 72 | 40 | 10 | 7.033 | 0.533 |

| | | | | | | | | |
|---|---|---|---|---|---|---|---|---|
| | % | 26.4 | 23.1 | 29.8 | 16.5 | 4.1 | | |
| Not passed | N =136 | 25 | 38 | 40 | 22 | 11 | | |
| | % | 18.4 | 27.9 | 29.4 | 16.2 | 8.1 | | |
| No exam in curricula | N =131 | 31 | 29 | 36 | 25 | 10 | | |
| | % | 23.7 | 22.1 | 27.5 | 19.1 | 7.6 | | |
| 9. Regular consumption of nuts contributes to the reduction of inflammation. | | | | | | | | |
| | | 1 | 2 | 3 | 4 | 5 | Stat. | p-value |
| Passed | N =242 | 8 | 36 | 110 | 56 | 32 | 10.664 | 0.221 |
| | % | 3.3 | 14.9 | 45.5 | 23.1 | 13.2 | | |
| Not passed | N =135 | 8 | 23 | 63 | 28 | 13 | | |
| | % | 5.9 | 17.0 | 46.7 | 20.7 | 9.6 | | |
| No exam in curricula | N =131 | 8 | 20 | 74 | 17 | 12 | | |
| | % | 6.1 | 15.3 | 56.5 | 13.0 | 9.2 | | |
| 10. Regular consumption of nuts contributes to the maintenance of normal endothelial function. | | | | | | | | |
| | | 1 | 2 | 3 | 4 | 5 | Stat. | p-value |
| Passed | N =242 | 4 | 21 | 94 | 81 | 42 | 18.935 | 0.015* |
| | % | 1.7 | 8.7 | 38.8 | 33.5 | 17.4 | | |
| Not passed | N =135 | 4 | 13 | 55 | 47 | 16 | | |
| | % | 3.0 | 9.6 | 40.7 | 34.8 | 11.9 | | |
| No exam in curricula | N =131 | 4 | 18 | 70 | 23 | 16 | | |
| | % | 3.1 | 13.7 | 53.4 | 17.6 | 12.2 | | |
| 11. Regular consumption of nuts can have a beneficial effect on the oxidative status in the body. | | | | | | | | |
| | | 1 | 2 | 3 | 4 | 5 | Stat. | p-value |
| Passed | N =242 | 0 | 18 | 65 | 86 | 73 | 24.498[f] | 0.002* |
| | % | 0.0 | 7.4 | 26.9 | 35.5 | 30.2 | | |
| Not passed | N =135 | 1 | 9 | 39 | 57 | 29 | | |
| | % | .7 | 6.7 | 28.9 | 42.2 | 21.5 | | |
| No exam in curricula | N =131 | 3 | 17 | 52 | 35 | 24 | | |
| | % | 2.3 | 13.0 | 39.7 | 26.7 | 18.3 | | |
| 12. It is important to know that nuts represent a source of protein in the diet. | | | | | | | | |

| | | 1 | 2 | 3 | 4 | 5 | Stat. | p-value |
|---|---|---|---|---|---|---|---|---|
| Passed | N =242 | 14 | 27 | 48 | 63 | 90 | 6.611 | 0.579 |
| | % | 5.8 | 11.2 | 19.8 | 26.0 | 37.2 | | |
| Not passed | N =136 | 8 | 16 | 23 | 46 | 43 | | |
| | % | 5.9 | 11.8 | 16.9 | 33.8 | 31.6 | | |
| No exam in curricula | N =131 | 11 | 14 | 30 | 29 | 47 | | |
| | % | 8.4 | 10.7 | 22.9 | 22.1 | 35.9 | | |
| 13. Nuts should be consumed regularly because it is a good source of unsaturated (healthy) fats in the diet. | | | | | | | | |
| | | 1 | 2 | 3 | 4 | 5 | Stat. | p-value |
| Passed | N =242 | 0 | 10 | 36 | 57 | 139 | 10.205 | 0.251 |
| | % | 0.0 | 4.1 | 14.9 | 23.6 | 57.4 | | |
| Not passed | N =136 | 1 | 8 | 22 | 41 | 64 | | |
| | % | .7 | 5.9 | 16.2 | 30.1 | 47.1 | | |
| No exam in curricula | N =131 | 2 | 7 | 27 | 35 | 60 | | |
| | % | 1.5 | 5.3 | 20.6 | 26.7 | 45.8 | | |
| 14. Nuts should be consumed regularly because it is a good source of essential fatty acids in the diet. | | | | | | | | |
| | | 1 | 2 | 3 | 4 | 5 | Stat. | p-value |
| Passed | N =242 | 1 | 11 | 44 | 69 | 117 | 19.354 | 0.013* |
| | % | .4 | 4.5 | 18.2 | 28.5 | 48.3 | | |
| Not passed | N =136 | 4 | 5 | 31 | 38 | 58 | | |
| | % | 2.9 | 3.7 | 22.8 | 27.9 | 42.6 | | |
| No exam in curricula | N =131 | 4 | 7 | 44 | 34 | 42 | | |
| | % | 3.1 | 5.3 | 33.6 | 26.0 | 32.1 | | |
| 15. Nuts can contribute to the preservation and improvement of health as a significant source of antioxidants. | | | | | | | | |
| | | 1 | 2 | 3 | 4 | 5 | Stat. | p-value |
| Passed | N =242 | 3 | 11 | 40 | 85 | 103 | 20.169 | 0.010* |
| | % | 1.2 | 4.5 | 16.5 | 35.1 | 42.6 | | |
| Not passed | N =135 | 2 | 7 | 25 | 47 | 54 | | |
| | % | 1.5 | 5.2 | 18.5 | 34.8 | 40.0 | | |
| No exam in | N =131 | 6 | 7 | 42 | 33 | 43 | | |

| | | | | | | | | |
|---|---|---|---|---|---|---|---|---|
| curricula | % | 4.6 | 5.3 | 32.1 | 25.2 | 32.8 | | |
| 16. Almonds are a good nutritional source of iron in the diet. | | | | | | | | |
| | | 1 | 2 | 3 | 4 | 5 | Stat. | p-value |
| Passed | N =242 | 15 | 37 | 83 | 55 | 52 | 9.537 | 0.299 |
| | % | 6.2 | 15.3 | 34.3 | 22.7 | 21.5 | | |
| Not passed | N =136 | 8 | 17 | 34 | 41 | 36 | | |
| | % | 5.9 | 12.5 | 25.0 | 30.1 | 26.5 | | |
| No exam in curricula | N =131 | 10 | 11 | 46 | 36 | 28 | | |
| | % | 7.6 | 8.4 | 35.1 | 27.5 | 21.4 | | |
| 17. Nuts should be recommended as a healthy snack in the diet. | | | | | | | | |
| | | 1 | 2 | 3 | 4 | 5 | Stat. | p-value |
| Passed | N =242 | 2 | 5 | 26 | 59 | 150 | 10.391[f] | 0.219 |
| | % | .8 | 2.1 | 10.7 | 24.4 | 62.0 | | |
| Not passed | N =136 | 1 | 5 | 13 | 40 | 77 | | |
| | % | .7 | 3.7 | 9.6 | 29.4 | 56.6 | | |
| No exam in curricula | N = 131 | 4 | 7 | 20 | 30 | 70 | | |
| | % | 3.1 | 5.3 | 15.3 | 22.9 | 53.4 | | |
| 18. The general population is not sufficiently informed about the health benefits of nuts. | | | | | | | | |
| | | 1 | 2 | 3 | 4 | 5 | Stat. | p-value |
| Passed | N =242 | 3 | 7 | 32 | 53 | 147 | 10.401[f] | 0.223 |
| | % | 1.2 | 2.9 | 13.2 | 21.9 | 60.7 | | |
| Not passed | N =136 | 1 | 6 | 14 | 28 | 87 | | |
| | % | .7 | 4.4 | 10.3 | 20.6 | 64.0 | | |
| No exam in curricula | N = 131 | 5 | 5 | 26 | 23 | 72 | | |
| | % | 3.8 | 3.8 | 19.8 | 17.6 | 55.0 | | |
| 19. One of the main barriers to the regular consumption of nuts is the high price. | | | | | | | | |
| | | 1 | 2 | 3 | 4 | 5 | Stat. | p-value |
| Passed | N =242 | 3 | 13 | 48 | 71 | 107 | 15.541 | 0.049 |
| | % | 1.2 | 5.4 | 19.8 | 29.3 | 44.2 | | |
| Not passed | N =136 | 5 | 10 | 11 | 46 | 64 | | |

| | | | | | | | | |
|---|---|---|---|---|---|---|---|---|
| | % | 3.7 | 7.4 | 8.1 | 33.8 | 47.1 | | |
| No exam in curricula | N =131 | 5 | 11 | 28 | 33 | 54 | | |
| | % | 3.8 | 8.4 | 21.4 | 25.2 | 41.2 | | |
| 20. I rarely consume nuts for fear of gaining weight. | | | | | | | | |
| | | 1 | 2 | 3 | 4 | 5 | Stat. | p-value |
| Passed | N = 242 | 153 | 34 | 29 | 17 | 9 | 12.755 | 0.121 |
| | % | 63.2 | 14.0 | 12.0 | 7.0 | 3.7 | | |
| Not passed | N = 136 | 71 | 24 | 24 | 10 | 7 | | |
| | % | 52.2 | 17.6 | 17.6 | 7.4 | 5.1 | | |
| No exam in curricula | N = 131 | 69 | 13 | 28 | 12 | 9 | | |
| | % | 52.7 | 9.9 | 21.4 | 9.2 | 6.9 | | |
| 21. I would try to consume nuts more often if recommended by a doctor, nutritionist or other health professional. | | | | | | | | |
| | | 1 | 2 | 3 | 4 | 5 | Stat. | p-value |
| Passed | N = 242 | 32 | 29 | 56 | 52 | 73 | 6.275 | 0.616 |
| | % | 13.2 | 12.0 | 23.1 | 21.5 | 30.2 | | |
| Not passed | N = 136 | 16 | 12 | 30 | 32 | 46 | | |
| | % | 11.8 | 8.8 | 22.1 | 23.5 | 33.8 | | |
| No exam in curricula | N = 131 | 22 | 15 | 35 | 19 | 40 | | |
| | % | 16.8 | 11.5 | 26.7 | 14.5 | 30.5 | | |
| 22. Nuts are one of the most common causes of allergic reactions to food. | | | | | | | | |
| | | 1 | 2 | 3 | 4 | 5 | Stat. | p-value |
| Passed | N = 242 | 7 | 20 | 47 | 73 | 95 | 42.558 | <0.001* |
| | % | 2.9 | 8.3 | 19.4 | 30.2 | 39.3 | | |
| Not passed | N = 136 | 10 | 17 | 31 | 50 | 28 | | |
| | % | 7.4 | 12.5 | 22.8 | 36.8 | 20.6 | | |
| No exam in curricula | N = 131 | 17 | 19 | 39 | 33 | 23 | | |
| | % | 13.0 | 14.5 | 29.8 | 25.2 | 17.6 | | |

### ***The influence of passing the nutrition-related exam (Bromatology) on the knowledge about nuts***

| | | Passed Bromatology (N=242) | | Not passed Bromatology (N=136) | | No exam in curricula (N=131) | | Test | |
|---|---|---|---|---|---|---|---|---|---|
| **Question** | **Answer** | **Correct answer (N)** | **Correct answer (N)** | **Correct answer (N)** | **Correct answer (N)** | **N** | **%** | **Stat.** | **p** |
| **1. In the dietary recommendations (nutrition pyramid), nuts are classified together with the group of foods:** | Cereals | 74 | 30.6 | 36 | 26.5 | 31 | 23.7 | 60.982 | <0.001* |
| | milk and milk products | 7 | 2.9 | 5 | 3.7 | 2 | 1.5 | | |
| | Fruits | 70 | 28.9 | 20 | 14.7 | 17 | 13.0 | | |
| | Vegetables | 4 | 1.7 | 0 | 0.0 | 0 | 0.0 | | |
| | *meat and eggs* | 36 | 14.9 | 18 | 13.2 | 13 | 9.9 | | |
| | Oils | 22 | 9.1 | 8 | 5.9 | 12 | 9.2 | | |
| | I do not know | 29 | 12.0 | 49 | 36.0 | 56 | 42.7 | | |
| **2. Nuts are an important food group in the Mediterranean diet:** | *Yes* | 175 | 72.3 | 74 | 54.4 | 51 | 38.9 | 55.695 | <0.001* |
| | No | 20 | 8.3 | 10 | 7.4 | 5 | 3.8 | | |
| | I do not know | 47 | 19.4 | 52 | 38.2 | 75 | 57.3 | | |
| **3. Nuts are an important food group in the DASH diet:** | *Yes* | 123 | 50.8 | 13 | 9.6 | 14 | 10.7 | 135.042 | <0.001* |
| | No | 17 | 7.0 | 1 | 0.7 | 0 | 0.0 | | |
| | I do not know | 102 | 42.1 | 121 | 89.7 | 117 | 89.3 | | |
| **4. Nuts are definitely not a source of which mineral in the diet?** | Selenium | 8 | 3.3 | 2 | 1.5 | 0 | 0.0 | 36.713 | <0.001* |
| | *Iodine* | 101 | 41.7 | 53 | 39.0 | 27 | 20.6 | | |
| | Magnesium | 20 | 8.3 | 5 | 3.7 | 6 | 4.6 | | |
| | Iron | 16 | 6.6 | 6 | 4.4 | 9 | 6.9 | | |
| | Zinc | 15 | 6.2 | 8 | 5.9 | 7 | 5.3 | | |
| | I do not know | 82 | 33.9 | 62 | 45.6 | 82 | 62.2 | | |
| **5. Nuts are a rich source of which vitamin in the diet?** | vitamin A | 12 | 5.0 | 5 | 3.7 | 7 | 5.3 | 75.897 | <0.001* |
| | vitamin C | 8 | 3.3 | 2 | 1.5 | 4 | 3.1 | | |
| | *vitamin E* | 138 | 57.0 | 35 | 25.7 | 27 | 20.6 | | |
| | vitamin D | 7 | 2.9 | 8 | 5.9 | 4 | 3.1 | | |
| | vitamin B12 | 35 | 14.5 | 28 | 20.6 | 25 | 19.1 | | |
| | I do not know | 42 | 17.4 | 58 | 42.6 | 64 | 48.9 | | |
| **6. Which of the nuts representatives is considered a good source of dietary selenium?** | Almond | 31 | 12.8 | 11 | 8.1 | 10 | 7.6 | 38.577 | <0.001* |
| | Hazelnut | 5 | 2.1 | 2 | 1.5 | 1 | 0.8 | | |
| | *Brazilian nut* | 71 | 29.3 | 29 | 21.3 | 21 | 16.0 | | |
| | Walnut | 18 | 7.4 | 6 | 4.4 | 2 | 1.5 | | |

| | | | | | | | | | |
|---|---|---|---|---|---|---|---|---|---|
| | Cashew | 39 | 16.1 | 13 | 9.6 | 17 | 13.0 | | |
| | I do not know | 78 | 32.2 | 75 | 55.1 | 80 | 61.1 | | |
| **7. Nuts are primarily a source of which polyunsaturated fatty acid?** | EPA | 34 | 14.0 | 4 | 2.9 | 1 | 0.8 | 98.091 | <0.001* |
| | DHA | 22 | 9.1 | 2 | 1.5 | 4 | 3.1 | | |
| | *linoleic acid* | 21 | 8.7 | 16 | 11.8 | 5 | 3.8 | | |
| | alpha-linolenic acid | 48 | 19.8 | 9 | 6.6 | 6 | 4.6 | | |
| | arachidonic acid | 14 | 5.8 | 17 | 12.5 | 4 | 3.1 | | |
| | I do not know | 103 | 42.6 | 88 | 64.7 | 111 | 84.7 | | |
| **8. Which of nuts has the highest content of saturated fatty acids and is therefore the least recommended in the diet compared to other stone fruits?** | Peanuts | 123 | 50.8 | 48 | 35.3 | 48 | 36.6 | 29.515 | 0.001* |
| | Almond | 14 | 5.8 | 9 | 6.6 | 7 | 5.3 | | |
| | Walnut | 16 | 6.6 | 5 | 3.7 | 2 | 1.5 | | |
| | Hazelnut | 3 | 1.2 | 2 | 1.5 | 3 | 2.3 | | |
| | *Cashew* | 30 | 12.4 | 12 | 8.8 | 14 | 10.7 | | |
| | I do not know | 56 | 23.1 | 60 | 44.1 | 57 | 43.5 | | |
| **9. There is an approved health claim for walnuts that reads: "Walnuts contribute to improving the elasticity of blood vessels." The stated health statement refers to the daily intake of walnuts in which of the stated quantities?** | 10g | 25 | 10.3 | 10 | 7.4 | 7 | 5.3 | 20.212 | 0.063 |
| | 15g | 37 | 15.3 | 27 | 19.9 | 12 | 9.2 | | |
| | *30g* | 63 | 26.0 | 23 | 16.9 | 26 | 19.8 | | |
| | 50g | 14 | 5.8 | 10 | 7.4 | 13 | 9.9 | | |
| | 100g | 6 | 2.5 | 3 | 2.2 | 2 | 1.5 | | |
| | more than 100g | 0 | 0.0 | 0 | 0.0 | 1 | 0.8 | | |
| | I do not know | 97 | 40.1 | 63 | 46.3 | 70 | 53.4 | | |
| **10. The usual thermal processing of nuts has an effect on their chemical composition and thus on their health effects, in which direction: :** | *Lowers* | 145 | 59.9 | 65 | 47.8 | 51 | 38.9 | 24.438 | <0.001* |
| | increases | 10 | 4.1 | 5 | 3.7 | 4 | 3.1 | | |
| | do not change | 33 | 13.6 | 15 | 11.0 | 16 | 12.2 | | |
| | I do not know | 54 | 22.3 | 51 | 37.5 | 60 | 45.8 | | |
| **11. Thermal processing of nuts impacts allergenicity, in which direction:** | Lowers | 59 | 24.4 | 25 | 18.4 | 12 | 9.2 | 31.073 | <0.001* |
| | increases | 19 | 7.9 | 11 | 8.1 | 9 | 6.9 | | |
| | *do not change* | 81 | 33.5 | 34 | 25.0 | 28 | 21.4 | | |
| | I do not know | 83 | 34.3 | 66 | 48.5 | 82 | 62.6 | | |
| **12. According to its** | *True* | 128 | 52.9 | 64 | 47.1 | 39 | 29.8 | 22.830 | <0.001* |

| | | | | | | | | | |
|---|---|---|---|---|---|---|---|---|---|
| **composition and health effects, peanuts can be counted as a nut, even though it belongs to the leguminous family:** | False | 19 | 7.9 | 13 | 9.6 | 8 | 6.1 | | |
| | I do not know | 95 | 39.3 | 59 | 43.3 | 84 | 64.1 | | |
| **13. By what characteristic does the chestnut significantly differ from other types of nuts?** | low content of mineral substances | 8 | 3.3 | 3 | 2.2 | 5 | 3.8 | 22.498 | 0.004* |
| | lower content of vitamin C | 10 | 4.1 | 4 | 2.9 | 4 | 3.1 | | |
| | higher energy value | 23 | 9.5 | 6 | 4.4 | 8 | 6.1 | | |
| | *higher carbohydrate content* | 75 | 31.0 | 25 | 18.4 | 21 | 16.0 | | |
| | I do not know | 126 | 52.1 | 98 | 72.1 | 93 | 71.0 | | |

***Correct answers are indicated in italic.***

***The influence of passing the nutrition-related exam (Nutrition) on attitudes regarding the health effects and nutritional importance of nuts***

| 1. Nuts represent a group of foods of exceptional nutritional value. | | | | | | | | |
|---|---|---|---|---|---|---|---|---|
| | | 1 | 2 | 3 | 4 | 5 | Stat. | p-value |
| Passed Nutrition | N = 187 | 1 | 4 | 28 | 50 | 104 | 7.147[f] | 0.500 |
| | % | .5 | 2.1 | 15.0 | 26.7 | 55.6 | | |
| Not passed | N = 181 | 1 | 5 | 21 | 63 | 91 | | |
| | % | .6 | 2.8 | 11.6 | 34.8 | 50.3 | | |
| Not in curricula | N = 140 | 1 | 5 | 26 | 36 | 72 | | |
| | % | .7 | 3.6 | 18.6 | 25.7 | 51.4 | | |
| 2. Regular consumption of nuts can have beneficial effects on health. | | | | | | | | |
| | | 1 | 2 | 3 | 4 | 5 | Stat. | p-value |
| Passed Nutrition | N = 187 | 0 | 6 | 14 | 52 | 115 | 19.412[f] | 0.006* |
| | % | 0.0 | 3.2 | 7.5 | 27.8 | 61.5 | | |
| Not passed | N = 181 | 0 | 2 | 22 | 51 | 106 | | |
| | % | 0.0 | 1.1 | 12.2 | 28.2 | 58.6 | | |
| Not in curricula | N = 140 | 1 | 8 | 26 | 41 | 64 | | |
| | % | .7 | 5.7 | 18.6 | 29.3 | 45.7 | | |
| 3. Consuming nuts contributes to the maintenance of normal lipid status. | | | | | | | | |
| | | 1 | 2 | 3 | 4 | 5 | Stat. | p-value |
| Passed Nutrition | N = 187 | 3 | 3 | 44 | 58 | 79 | 18.406 | 0.018* |
| | % | 1.6 | 1.6 | 23.5 | 31.0 | 42.2 | | |
| Not passed | N = 180 | 1 | 10 | 43 | 55 | 71 | | |
| | % | .6 | 5.6 | 23.9 | 30.6 | 39.4 | | |
| Not in curricula | N = 140 | 2 | 8 | 54 | 32 | 44 | | |
| | % | 1.4 | 5.7 | 38.6 | 22.9 | 31.4 | | |
| 4. Regular and moderate consumption of nuts contributes to reducing the risk of developing cardiovascular diseases. | | | | | | | | |
| | | 1 | 2 | 3 | 4 | 5 | Stat. | p-value |
| Passed Nutrition | N = 187 | 0 | 8 | 36 | 67 | 76 | 18.187 | 0.020* |
| | % | 0.0 | 4.3 | 19.3 | 35.8 | 40.6 | | |
| Not passed | N = 181 | 0 | 5 | 46 | 56 | 74 | | |

| | | | | | | | | |
|---|---|---|---|---|---|---|---|---|
| | % | 0.0 | 2.8 | 25.4 | 30.9 | 40.9 | | |
| Not in curricula | N = 140 | 4 | 9 | 37 | 46 | 44 | | |
| | % | 2.9 | 6.4 | 26.4 | 32.9 | 31.4 | | |
| 5. Regular and moderate consumption of nuts contributes to reducing the risk of developing diabetes. | | | | | | | | |
| | | 1 | 2 | 3 | 4 | 5 | Stat. | p-value |
| Passed Nutrition | N = 187 | 3 | 17 | 48 | 66 | 53 | 9.374 | 0.312 |
| | % | 1.6 | 9.1 | 25.7 | 35.3 | 28.3 | | |
| Not passed | N = 181 | 3 | 18 | 55 | 53 | 52 | | |
| | % | 1.7 | 9.9 | 30.4 | 29.3 | 28.7 | | |
| Not in curricula | N = 140 | 4 | 17 | 52 | 33 | 34 | | |
| | % | 2.9 | 12.1 | 37.1 | 23.6 | 24.3 | | |
| 6. Regular and moderate consumption of nuts contributes to reducing the risk of cancer. | | | | | | | | |
| | | 1 | 2 | 3 | 4 | 5 | Stat. | p-value |
| Passed Nutrition | N = 187 | 7 | 30 | 67 | 50 | 33 | 7.488 | 0.485 |
| | % | 3.7 | 16.0 | 35.8 | 26.7 | 17.6 | | |
| Not passed | N = 181 | 6 | 25 | 63 | 41 | 46 | | |
| | % | 3.3 | 13.8 | 34.8 | 22.7 | 25.4 | | |
| Not in curricula | N = 140 | 6 | 30 | 49 | 29 | 26 | | |
| | % | 4.3 | 21.4 | 35.0 | 20.7 | 18.6 | | |
| 7. Regular and moderate consumption of nuts contributes to an increase in total cholesterol. | | | | | | | | |
| | | 1 | 2 | 3 | 4 | 5 | Stat. | p-value |
| Passed Nutrition | N = 187 | 28 | 50 | 56 | 34 | 19 | 4.345 | 0.825 |
| | % | 15.0 | 26.7 | 29.9 | 18.2 | 10.2 | | |
| Not passed | N = 181 | 22 | 53 | 67 | 24 | 15 | | |
| | % | 12.2 | 29.3 | 37.0 | 13.3 | 8.3 | | |
| Not in curricula | N = 140 | 17 | 37 | 50 | 22 | 14 | | |
| | % | 12.1 | 26.4 | 35.7 | 15.7 | 10.0 | | |
| 8. Consuming nuts several times a week contributes to an increase in body weight. | | | | | | | | |
| | | 1 | 2 | 3 | 4 | 5 | Stat. | p-value |
| Passed | N = 187 | 48 | 41 | 55 | 34 | 9 | 5.336 | 0.721 |

| | | | | | | | | |
|---|---|---|---|---|---|---|---|---|
| Nutrition | % | 25.7 | 21.9 | 29.4 | 18.2 | 4.8 | | |
| Not passed | N = 181 | 39 | 51 | 54 | 26 | 11 | | |
| | % | 21.5 | 28.2 | 29.8 | 14.4 | 6.1 | | |
| Not in curricula | N = 140 | 33 | 30 | 39 | 27 | 11 | | |
| | % | 23.6 | 21.4 | 27.9 | 19.3 | 7.9 | | |
| 9. Regular consumption of nuts contributes to the reduction of inflammation. | | | | | | | | |
| | | 1 | 2 | 3 | 4 | 5 | Stat. | p-value |
| Passed Nutrition | N = 187 | 2 | 26 | 86 | 46 | 27 | 19.527 | 0.012* |
| | % | 1.1 | 13.9 | 46.0 | 24.6 | 14.4 | | |
| Not passed | N = 180 | 11 | 32 | 86 | 36 | 15 | | |
| | % | 6.1 | 17.8 | 47.8 | 20.0 | 8.3 | | |
| Not in curricula | N = 140 | 11 | 21 | 75 | 18 | 15 | | |
| | % | 7.9 | 15.0 | 53.6 | 12.9 | 10.7 | | |
| 10. Regular consumption of nuts contributes to the maintenance of normal endothelial function. | | | | | | | | |
| | | 1 | 2 | 3 | 4 | 5 | Stat. | p-value |
| Passed Nutrition | N = 187 | 4 | 19 | 65 | 66 | 33 | 21.721 | 0.005* |
| | % | 2.1 | 10.2 | 34.8 | 35.3 | 17.6 | | |
| Not passed | N = 180 | 4 | 16 | 78 | 61 | 21 | | |
| | % | 2.2 | 8.9 | 43.3 | 33.9 | 11.7 | | |
| Not in curricula | N = 140 | 4 | 17 | 76 | 23 | 20 | | |
| | % | 2.9 | 12.1 | 54.3 | 16.4 | 14.3 | | |
| 11. Regular consumption of nuts can have a beneficial effect on the oxidative status in the body. | | | | | | | | |
| | | 1 | 2 | 3 | 4 | 5 | Stat. | p-value |
| Passed Nutrition | N = 187 | 1 | 12 | 56 | 67 | 51 | 14.363 | 0.073 |
| | % | .5 | 6.4 | 29.9 | 35.8 | 27.3 | | |
| Not passed | N = 180 | 1 | 14 | 47 | 73 | 45 | | |
| | % | .6 | 7.8 | 26.1 | 40.6 | 25.0 | | |
| Not in curricula | N = 140 | 2 | 18 | 53 | 37 | 30 | | |
| | % | 1.4 | 12.9 | 37.9 | 26.4 | 21.4 | | |
| 12. It is important to know that nuts represent a source of protein in the diet. | | | | | | | | |

| | | 1 | 2 | 3 | 4 | 5 | Stat. | p-value |
|---|---|---|---|---|---|---|---|---|
| Passed Nutrition | N = 187 | 12 | 19 | 42 | 51 | 63 | 7.067 | 0.529 |
| | % | 6.4 | 10.2 | 22.5 | 27.3 | 33.7 | | |
| Not passed | N = 181 | 9 | 24 | 28 | 54 | 66 | | |
| | % | 5.0 | 13.3 | 15.5 | 29.8 | 36.5 | | |
| Not in curricula | N = 140 | 12 | 14 | 31 | 32 | 51 | | |
| | % | 8.6 | 10.0 | 22.1 | 22.9 | 36.4 | | |
| 13. Nuts should be consumed regularly because it is a good source of unsaturated (healthy) fats in the diet. | | | | | | | | |
| | | 1 | 2 | 3 | 4 | 5 | Stat. | p-value |
| Passed Nutrition | N = 187 | 0 | 9 | 29 | 47 | 102 | 4.496 | 0.810 |
| | % | 0.0 | 4.8 | 15.5 | 25.1 | 54.5 | | |
| Not passed | N = 181 | 1 | 8 | 30 | 48 | 94 | | |
| | % | .6 | 4.4 | 16.6 | 26.5 | 51.9 | | |
| Not in curricula | N = 140 | 2 | 8 | 26 | 38 | 66 | | |
| | % | 1.4 | 5.7 | 18.6 | 27.1 | 47.1 | | |
| 14. Nuts should be consumed regularly because it is a good source of essential fatty acids in the diet | | | | | | | | |
| | | 1 | 2 | 3 | 4 | 5 | Stat. | p-value |
| Passed Nutrition | N = 187 | 2 | 6 | 35 | 57 | 87 | 14.084 | 0.080 |
| | % | 1.1 | 3.2 | 18.7 | 30.5 | 46.5 | | |
| Not passed | N = 181 | 4 | 9 | 38 | 48 | 82 | | |
| | % | 2.2 | 5.0 | 21.0 | 26.5 | 45.3 | | |
| Not in curricula | N = 140 | 3 | 8 | 46 | 36 | 47 | | |
| | % | 2.1 | 5.7 | 32.9 | 25.7 | 33.6 | | |
| 15. Nuts can contribute to the preservation and improvement of health as a significant source of antioxidants. | | | | | | | | |
| | | 1 | 2 | 3 | 4 | 5 | Stat. | p-value |
| Passed Nutrition | N = 187 | 4 | 7 | 32 | 68 | 76 | 16.059 | 0.042* |
| | % | 2.1 | 3.7 | 17.1 | 36.4 | 40.6 | | |
| Not passed | N = 180 | 1 | 10 | 34 | 60 | 75 | | |
| | % | .6 | 5.6 | 18.9 | 33.3 | 41.7 | | |
| Not in curricula | N = 140 | 6 | 8 | 41 | 36 | 49 | | |

| | | | | | | | | |
|---|---|---|---|---|---|---|---|---|
| | % | 4.3 | 5.7 | 29.3 | 25.7 | 35.0 | | |
| 16. Almonds are a good nutritional source of iron in the diet. | | | | | | | | |
| | | 1 | 2 | 3 | 4 | 5 | Stat. | p-value |
| Passed Nutrition | N = 187 | 13 | 27 | 63 | 42 | 42 | 3.934 | 0.863 |
| | % | 7.0 | 14.4 | 33.7 | 22.5 | 22.5 | | |
| Not passed | N = 181 | 10 | 23 | 52 | 53 | 43 | | |
| | % | 5.5 | 12.7 | 28.7 | 29.3 | 23.8 | | |
| Not in curricula | N = 140 | 10 | 15 | 47 | 37 | 31 | | |
| | % | 7.1 | 10.7 | 33.6 | 26.4 | 22.1 | | |
| 17. Nuts should be recommended as a healthy snack in the diet. | | | | | | | | |
| | | 1 | 2 | 3 | 4 | 5 | Stat. | p-value |
| Passed Nutrition | N = 187 | 2 | 5 | 20 | 47 | 113 | 8.503[f] | 0.375 |
| | % | 1.1 | 2.7 | 10.7 | 25.1 | 60.4 | | |
| Not passed | N = 181 | 1 | 5 | 17 | 49 | 109 | | |
| | % | .6 | 2.8 | 9.4 | 27.1 | 60.2 | | |
| Not in curricula | N = 140 | 4 | 7 | 22 | 33 | 74 | | |
| | % | 2.9 | 5.0 | 15.7 | 23.6 | 52.9 | | |
| 18. The general population is not sufficiently informed about the health benefits of nuts. | | | | | | | | |
| | | 1 | 2 | 3 | 4 | 5 | Stat. | p-value |
| Passed Nutrition | N = 187 | 4 | 6 | 25 | 39 | 113 | 8.343[f] | 0.394 |
| | % | 2.1 | 3.2 | 13.4 | 20.9 | 60.4 | | |
| Not passed | N = 181 | 1 | 8 | 21 | 42 | 109 | | |
| | % | .6 | 4.4 | 11.6 | 23.2 | 60.2 | | |
| Not in curricula | N = 140 | 4 | 4 | 26 | 22 | 84 | | |
| | % | 2.9 | 2.9 | 18.6 | 15.7 | 60.0 | | |
| 19. One of the main barriers to the regular consumption of nuts is the high price. | | | | | | | | |
| | | 1 | 2 | 3 | 4 | 5 | Stat. | p-value |
| Passed Nutrition | N = 187 | 3 | 10 | 35 | 56 | 83 | 9.058 | 0.337 |
| | % | 1.6 | 5.3 | 18.7 | 29.9 | 44.4 | | |
| Not passed | N = 181 | 6 | 11 | 23 | 60 | 81 | | |

| | | | | | | | | |
|---|---|---|---|---|---|---|---|---|
| | % | 3.3 | 6.1 | 12.7 | 33.1 | 44.8 | | |
| Not in curricula | N = 140 | 4 | 13 | 29 | 33 | 61 | | |
| | % | 2.9 | 9.3 | 20.7 | 23.6 | 43.6 | | |
| 20. I rarely consume nuts for fear of gaining weight. | | | | | | | | |
| | | 1 | 2 | 3 | 4 | 5 | Stat. | p-value |
| Passed Nutrition | N = 187 | 113 | 26 | 27 | 15 | 6 | 11.288 | 0.186 |
| | % | 60.4 | 13.9 | 14.4 | 8.0 | 3.2 | | |
| Not passed | N = 181 | 106 | 31 | 25 | 10 | 9 | | |
| | % | 58.6 | 17.1 | 13.8 | 5.5 | 5.0 | | |
| Not in curricula | N = 140 | 73 | 14 | 29 | 14 | 10 | | |
| | % | 52.1 | 10.0 | 20.7 | 10.0 | 7.1 | | |
| 21. I would try to consume nuts more often if recommended by a doctor, nutritionist or other health professional. | | | | | | | | |
| | | 1 | 2 | 3 | 4 | 5 | Stat. | p-value |
| Passed Nutrition | N = 187 | 26 | 22 | 46 | 40 | 53 | 4.638 | 0.795 |
| | % | 13.9 | 11.8 | 24.6 | 21.4 | 28.3 | | |
| Not passed | N = 181 | 23 | 16 | 40 | 41 | 61 | | |
| | % | 12.7 | 8.8 | 22.1 | 22.7 | 33.7 | | |
| Not in curricula | N = 140 | 21 | 17 | 35 | 22 | 45 | | |
| | % | 15.0 | 12.1 | 25.0 | 15.7 | 32.1 | | |
| 22. Nuts are one of the most common causes of allergic reactions to food. | | | | | | | | |
| | | 1 | 2 | 3 | 4 | 5 | Stat. | p-value |
| Passed Nutrition | N = 187 | 10 | 19 | 33 | 56 | 69 | 25.139 | 0.001* |
| | % | 5.3 | 10.2 | 17.6 | 29.9 | 36.9 | | |
| Not passed | N = 181 | 9 | 17 | 40 | 67 | 48 | | |
| | % | 5.0 | 9.4 | 22.1 | 37.0 | 26.5 | | |
| Not in curricula | N = 140 | 15 | 20 | 43 | 33 | 29 | | |
| | % | 10.7 | 14.3 | 30.7 | 23.6 | 20.7 | | |

*The influence of passing the Nutrition-related exam (Nutrition) on knowledge about nuts*

| | | Passed (N=187) | | Not passed (N=181) | | No exam in curricula (N=140) | | Test | |
|---|---|---|---|---|---|---|---|---|---|
| **Question** | **Answer** | **N** | **%** | **N** | **%** | **N** | **%** | **Stat.** | **p** |
| **1. In the dietary recommendations (nutrition pyramid), nuts are classified together with the group of foods:** | Cereals | 59 | 31.6 | 47 | 26.0 | 35 | 25.0 | 47.260 | <0.001* |
| | milk and milk products | 9 | 4.8 | 3 | 1.7 | 2 | 1.4 | | |
| | Fruits | 51 | 27.3 | 36 | 19.9 | 20 | 14.3 | | |
| | Vegetables | 2 | 1.1 | 2 | 1.1 | 0 | 0.0 | | |
| | meat and eggs | 26 | 13.9 | 29 | 16.0 | 12 | 8.6 | | |
| | Oils | 18 | 9.6 | 11 | 6.1 | 13 | 9.3 | | |
| | I do not know | 22 | 11.8 | 53 | 29.3 | 58 | 41.4 | | |
| **2. Nuts are an important food group in the Mediterranean diet:** | Yes | 135 | 72.2 | 106 | 58.6 | 58 | 41.4 | 53.314 | <0.001* |
| | No | 20 | 10.7 | 10 | 5.5 | 5 | 3.6 | | |
| | I do not know | 32 | 17.1 | 65 | 35.9 | 77 | 55.0 | | |
| **3. Nuts are an important food group in the DASH diet:** | Yes | 110 | 58.8 | 23 | 12.7 | 17 | 12.1 | 172.271 | <0.001* |
| | No | 18 | 9.6 | 0 | 0.0 | 0 | 0.0 | | |
| | I do not know | 59 | 31.6 | 158 | 87.3 | 123 | 87.9 | | |
| **4. Nuts definitely are not a source of which mineral in the diet?** | Selenium | 5 | 2.7 | 4 | 2.2 | 1 | 0.7 | 38.506 | <0.001* |
| | Iodine | 78 | 41.7 | 74 | 40.9 | 28 | 20.0 | | |
| | Magnesium | 19 | 10.2 | 6 | 3.3 | 6 | 4.3 | | |
| | Iron | 11 | 5.9 | 9 | 5.0 | 11 | 7.9 | | |
| | Zinc | 12 | 6.4 | 10 | 5.5 | 8 | 5.7 | | |
| | I do not know | 62 | 33.2 | 78 | 43.1 | 86 | 61.4 | | |
| **5. Nuts are a rich source of which vitamin in the diet?** | vitamin A | 10 | 5.3 | 8 | 4.4 | 6 | 4.3 | 60.534 | <0.001* |
| | vitamin C | 7 | 13.4 | 3 | 1.7 | 4 | 2.9 | | |
| | vitamin E | 107 | 57.2 | 63 | 34.8 | 30 | 21.4 | | |
| | vitamin D | 7 | 13.4 | 8 | 4.4 | 4 | 2.9 | | |
| | vitamin B12 | 25 | 13.4 | 36 | 19.9 | 26 | 18.6 | | |
| | I do not know | 31 | 16.6 | 63 | 34.8 | 70 | 50.0 | | |
| **6. Which of the nuts** | Almond | 23 | 12.3 | 18 | 9.9 | 11 | 7.9 | 27.946 | <0.001* |

| | | | | | | | | | |
|---|---|---|---|---|---|---|---|---|---|
| **representatives is considered a good source of dietary selenium?** | Hazelnut | 3 | 1.6 | 4 | 2.2 | 1 | 0.7 | | |
| | Brazilian nut | 54 | 28.9 | 44 | 24.3 | 23 | 16.4 | | |
| | Walnut | 13 | 7.0 | 11 | 6.1 | 2 | 1.4 | | |
| | Cashew | 31 | 16.6 | 19 | 10.5 | 19 | 13.6 | | |
| | I do not know | 63 | 33.7 | 85 | 47.0 | 84 | 60.0 | | |
| **7. Nuts are primarily a source of which polyunsaturated fatty acid?** | EPA | 31 | 16.6 | 6 | 3.3 | 2 | 1.4 | 99.804 | <0.001* |
| | DHA | 18 | 9.6 | 5 | 2.8 | 5 | 3.6 | | |
| | linoleic acid | 18 | 9.6 | 19 | 10.5 | 5 | 3.6 | | |
| | alpha-linolenic acid | 38 | 20.3 | 20 | 11.0 | 5 | 3.6 | | |
| | arachidonic acid | 10 | 5.3 | 20 | 11.0 | 5 | 3.6 | | |
| | I do not know | 72 | 38.5 | 111 | 61.3 | 118 | 84.3 | | |
| **8. Which of nuts has the highest content of saturated fatty acids and is therefore the least recommended in the diet compared to other stone fruits?** | Peanuts | 98 | 52.4 | 73 | 40.3 | 48 | 34.3 | 31.079 | 0.001* |
| | Almond | 9 | 4.8 | 12 | 6.6 | 9 | 6.4 | | |
| | Walnut | 13 | 7.0 | 7 | 3.9 | 3 | 2.1 | | |
| | Hazelnut | 2 | 1.1 | 3 | 1.7 | 3 | 2.1 | | |
| | Cashew | 26 | 13.9 | 13 | 7.2 | 17 | 12.1 | | |
| | I do not know | 39 | 20.9 | 73 | 40.3 | 60 | 42.9 | | |
| **9. There is an approved health claim for walnuts that reads: "Walnuts contribute to improving the elasticity of blood vessels." The stated health statement refers to the daily intake of walnuts in which of the stated quantities?** | 10g | 17 | 9.1 | 17 | 9.4 | 8 | 5.7 | 20.221 | 0.063 |
| | 15g | 27 | 14.4 | 37 | 20.4 | 12 | 8.6 | | |
| | 30g | 50 | 26.7 | 34 | 18.8 | 28 | 20.0 | | |
| | 50g | 11 | 5.9 | 13 | 7.2 | 13 | 9.3 | | |
| | 100g | 4 | 2.1 | 5 | 2.8 | 2 | 1.4 | | |
| | more than 100g | 0 | 0.0 | 0 | 0.0 | 1 | 0.7 | | |
| | I do not know | 78 | 41.7 | 75 | 41.4 | 76 | 54.3 | | |
| **10. Thermal processing of nuts impacts health effect, in which direction:** | Lowers | 115 | 61.5 | 94 | 51.9 | 52 | 37.1 | 25.917 | <0.001* |
| | Poincreases | 9 | 4.8 | 6 | 3.3 | 4 | 2.9 | | |
| | do not change | 23 | 12.3 | 22 | 12.2 | 19 | 13.6 | | |
| | I do not know | 40 | 21.4 | 59 | 32.6 | 65 | 46.4 | | |
| **11. Thermal processing of nuts impacts allergenicity,** | Lowers | 52 | 27.8 | 31 | 17.1 | 13 | 9.3 | 32.174 | <0.001* |
| | Poincreases | 15 | 8.0 | 14 | 7.7 | 10 | 7.1 | | |

| | | | | | | | | | |
|---|---|---|---|---|---|---|---|---|---|
| **in which direction:** | do not change | 60 | 32.1 | 51 | 28.2 | 32 | 22.9 | | |
| | I do not know | 60 | 32.1 | 85 | 47.0 | 85 | 60.7 | | |
| **12. According to its composition and health effects, peanuts can be counted as a nut, even though it belongs to the leguminous family:** | True | 99 | 52.9 | 89 | 49.2 | 43 | 30.7 | 29.766 | <0.001* |
| | False | 21 | 11.2 | 13 | 7.2 | 6 | 4.3 | | |
| | I do not know | 67 | 35.8 | 79 | 43.6 | 91 | 65.0 | | |
| **13. By what characteristic does the chestnut significantly differ from other types of nuts?** | low content of mineral substances | 7 | 3.7 | 4 | 2.2 | 5 | 3.6 | 21.723 | 0.005* |
| | lower content of vitamin C | 9 | 4.8 | 4 | 2.2 | 5 | 3.6 | | |
| | higher energy value | 19 | 10.2 | 10 | 5.5 | 8 | 5.7 | | |
| | higher carbohydrate content | 58 | 31.0 | 42 | 23.2 | 21 | 15.0 | | |
| | I do not know | 94 | 50.3 | 121 | 66.9 | 101 | 72.1 | | |
